# Bovine sperm-oviduct interactions are characterized by specific sperm behaviour, ultrastructure and tubal reactions which are impacted by sex sorting

**DOI:** 10.1038/s41598-020-73592-1

**Published:** 2020-10-05

**Authors:** Miguel Camara Pirez, Heather Steele, Sven Reese, Sabine Kölle

**Affiliations:** 1 School of Medicine, Health Sciences Centre, University College Dublin (UCD), Dublin, Ireland; 2 School of Veterinary Medicine, Institute of Veterinary Anatomy, Histology and Embryology, LMU, Munich, Germany

**Keywords:** Reproductive biology, Germ cells

## Abstract

To date sperm-oviduct interactions have largely been investigated under in vitro conditions. Therefore we set out to characterize the behaviour of bovine spermatozoa within the sperm reservoir under near in vivo conditions and in real-time using a novel live cell imaging technology and a newly established fluorescent sperm binding assay. Sperm structure and tubal reactions after sperm binding were analysed using scanning and transmission electron microscopy and histochemistry. As a model to specify the impact of stress on sperm-oviduct interactions, frozen-thawed conventional and sex-sorted spermatozoa from the same bulls (n = 7) were co-incubated with oviducts obtained from cows immediately after slaughter. Our studies revealed that within the oviductal sperm reservoir agile (bound at a tangential angle of about 30°, actively beating undulating tail), lagging (bound at a lower angle, reduced tail movement), immotile (absence of tail movement) and hyperactivated (whip-like movement of tail) spermatozoa occur, the prevalence of which changes in a time-dependent pattern. After formation of the sperm reservoir, tubal ciliary beat frequency is significantly increased (*p* = 0.022) and the epithelial cells show increased activity of endoplasmic reticula. After sex sorting, spermatozoa occasionally display abnormal movement patterns characterized by a 360° rotating head and tail. Sperm binding in the oviduct is significantly reduced (*p* = 0.008) following sexing. Sex-sorted spermatozoa reveal deformations in the head, sharp bends in the tail and a significantly increased prevalence of damaged mitochondria (*p* < 0.001). Our results imply that the oviductal cells specifically react to the binding of spermatozoa, maintaining sperm survival within the tubal reservoir. The sex-sorting process, which is associated with mechanical, chemical and time stress, impacts sperm binding to the oviduct and mitochondrial integrity affecting sperm motility and function.

## Introduction

As soon as spermatozoa enter the oviduct, they form a sperm reservoir by binding with their head to the ciliated cells of the oviductal epithelium. Under in vivo conditions, the majority of spermatozoa are bound to the isthmus (as this is the first part sperm encounter), however, spermatozoa also bind to the ampulla^1–3^. The presence of a sperm reservoir has been shown in mice, hamsters, pigs, cows and horses^4–8^. Through binding to the oviduct, spermatozoa are able to maintain their motility and their fertilizing capacity for 3–4 days (most mammals), for months (bats) or even years (snakes)^2,8–12^. Sperm binding is managed by the presence of species-specific carbohydrate moieties on the cilia, such as fucose in the cow, galactose in the horse, mannose/Lewis^X^ trisaccharide in the pig and sialic acid in the hamster^1,13–17^. In bulls and rams, these carbohydrate moieties on the cilia bind to specific sperm binder proteins (BSPs) which are located on the sperm head and are also involved in sperm capacitation and sperm detachment from the reservoir^18,19^. After sperm binding, gene and protein expression in the oviduct is altered resulting in changes of the composition of the oviductal fluid raising the question whether synthesis of proteins and carbohydrates is changed after sperm binding^20,21^. The composition of the tubal fluid is also modulated by hormonal changes initiating the synthesis of mucopolysaccharides and enzymes in the tubal secretory cells during ovulation^22^. In pigs, sperm binding may induce sex-specific changes in gene expression of oviductal cells in vivo with 501 transcripts known to be differentially expressed between oviductal epithelia incubated with X or Y-bearing spermatozoa. The majority of these transcripts are involved in signal transduction and immune response and are down-regulated in oviductal epithelia following contact with X-bearing spermatozoa^23^. In in vitro cell cultures, sulfated glycoconjugates and disulfide reductants have been shown to be involved in sperm release^24–27^. Under in vivo conditions, the changing concentrations of estrogen and progesterone are involved in the formation of the bovine sperm reservoir and the timely release of capacitated spermatozoa^28^. Recently the release of porcine sperm from the sperm reservoir has also been shown to be stimulated by progesterone and to require CatSper^29^.

Overall sperm-oviduct interactions are of physical and molecular nature. Physical interactions include the swimming responses of the spermatozoa to the microarchitecture of the inner tubal wall and to the flow of the oviductal fluid. Molecular interactions include the communication of sperm surface molecules to the receptors on the inner lining of the oviduct^30^. Every type of stress is prone to affect these interactions^31^. To elucidate stress-induced effects on sperm-oviduct interactions, we included the effect of sex-sorted spermatozoa in the study. During the sorting process spermatozoa undergo physical stress, chemical stress (fluorescent labelling), as well as stress caused by dilution and prolonged time of sperm handling^32^.

Sex-sorting of bovine spermatozoa has been introduced to control sex ratios in cattle in order to optimize food production, livestock management and genetic selection^33^. The most widely used method for sperm sex sorting is fluorescence-activated cell sorting (FACS)^34^. Since its inception, sperm sorting has not only successfully been applied in various breeds of cattle but also in sheep, horses, pigs, dogs, bottlenose dolphins and gorillas^35–43^. Due to ethical reasons, the use of sexed sperm in humans is extremely limited and restricted to families with a history of X-linked diseases such as hemophilia or Duchenne’s muscular dystrophy^44^. Sex sorting utilizes the difference in the DNA content of the sex chromosomes to accurately separate the spermatozoa into two populations. The spermatozoa are labelled with the fluorescent DNA-binding dye Hoechst 33,342, passed through a flow chamber, exposed to a UV laser and are separated using magnetic field exposure^45^.

There are a number of advantages to sex sorting spermatozoa. The process enables the separation of X- and Y-bearing spermatozoa with 90% accuracy, allows farmers to maintain their herds without having to buy cows from other sources and increases herd milk production through the manipulation of sex ratios^33,46^. However, sorting takes approximately 50 h to sort through a single bull ejaculate (sorting speed: 30,000 sperm/s) and the resulting straws are more expensive than unsorted straws^46,47^. Further to that, there is a 7.5-fold decrease in the sperm concentration of sexed straws as sperm are diluted due to sorting and centrifugation and 50% of the sperm are discarded^48^. But the biggest obstacle to the widespread use of sexed spermatozoa in the dairy industry is the decrease in fertility of sperm after sex sorting. Decreases in conception rates as high as 25% have been recorded in sexed spermatozoa and 2–3 sexed straws may be required before fertilization is achieved^49,50^. Consequently, the use of sexed spermatozoa is only recommended in heifers because conceptions rates decrease further in cows^51^.

One possible cause for the reduced conception rates is the altered sperm morphokinetics following sex-sorting. Computer-assisted semen analysis (CASA) of conventional and sexed spermatozoa of the same bulls reveal significantly reduced prevalence of fast and slow progressive spermatozoa as well as of hyperactivated spermatozoa^52^. In regard to molecular alterations following sorting, stallion spermatozoa show increased levels of reactive oxygen species (ROS), increased sperm membrane permeability, increased DNA fragmentation and reduced intracellular ATP levels^53–56^. Further detrimental effects of sex-sorting include decreased motility and higher percentages of acrosomally exocytosed sperm after thawing, which have been documented in rams^57,58^. Similarly, bull spermatozoa display decreased amounts of acrosome-intact spermatozoa, reduced mitochondrial membrane potential, decreased stability of the plasma membrane affecting capacitation as well as reduced motility and longevity after sorting^31,59,60^.

Overall, the duration of the sorting process, the staining with a fluorescent dye and the mechanical stress of the sorting process exert numerous effects on spermatozoa, most of which are largely unknown. The precise knowledge of the induced alterations in spermatozoa are the prerequisites for establishing novel concepts for improving the sorting procedure, allowing for the widespread use of this technology. Further to that, it provides valuable insights for human medicine in regard to how different kinds of stress affect successful sperm-oviduct interactions and sperm survival in the female genital tract.

Therefore, the purpose of this study was to elucidate sperm-oviduct interactions and to characterize stress-related alterations under near in vivo conditions and in real time using a digital live cell imaging technology established in our research group. These results were correlated to the response of the oviduct to sperm binding, which was analysed by histochemistry, and to the morphological changes of sperm and tubal epithelium as seen by scanning and transmission electron microscopy.

## Results

### Spermatozoa behave in a specific manner in the tubal sperm reservoir which partially changes after sex sorting

**Figure 1:**
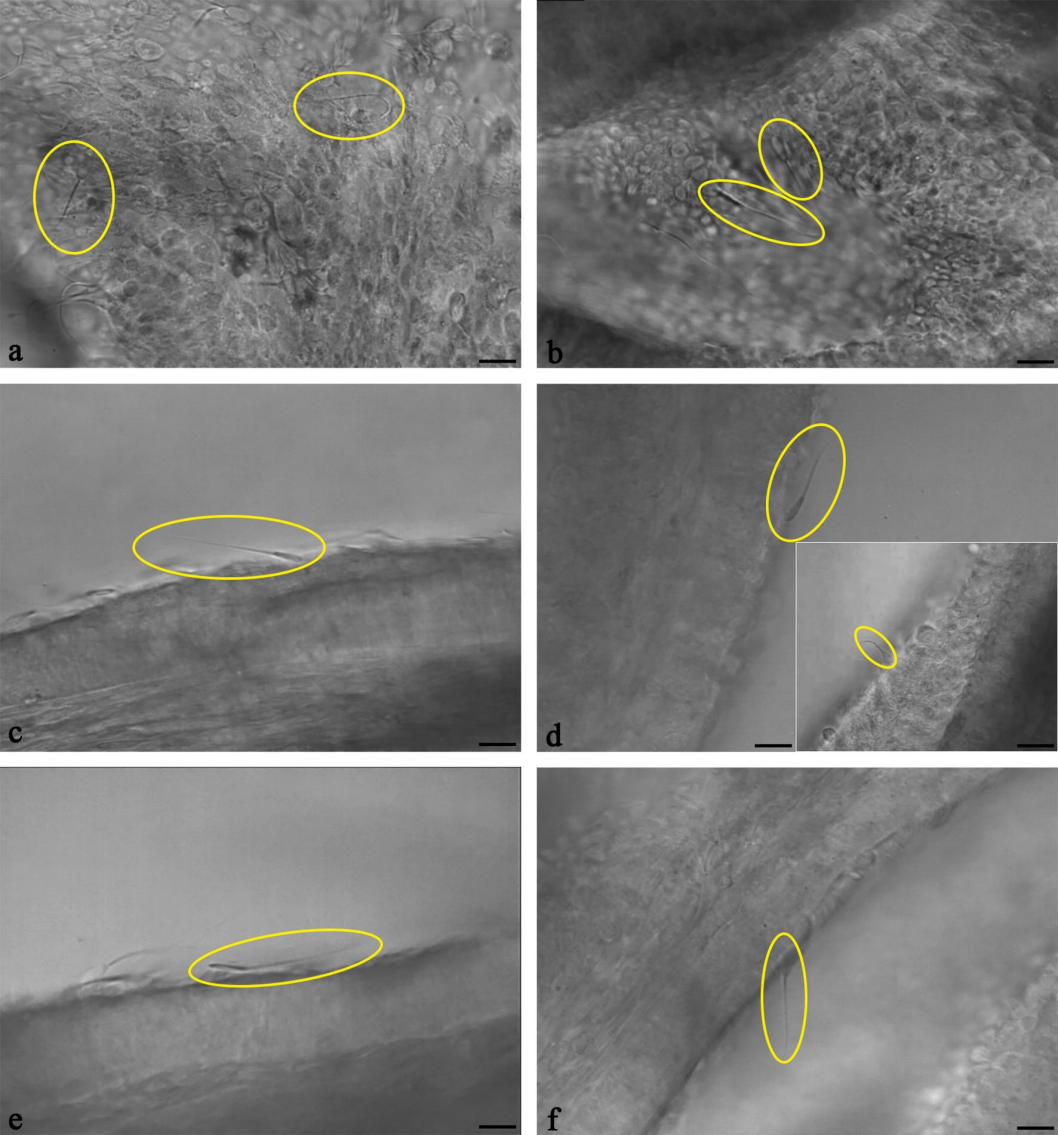
Live cell imaging of bovine spermatozoa forming the reservoir in the ampulla (n = 7 bulls, n = 14 ampullae). (**a**–**b**) Spermatozoa (circles) bind to the ampullar cilia both in conventional (**a**) and sexed (**b**) spermatozoa (see Movies 1 and 2). (**c**) Agile spermatozoa (circle, conventional spermatozoon) bind to the cilia at a tangential angle of about 30° with an actively beating, undulating tail (see Movie 3, spermatozoon no 2). (**d**) Lagging spermatozoa (circle, sexed spermatozoon) bind to the cilia in a decreased angle and reveal reduced tail movement (see Movie 4). Sexed spermatozoa occasionally show 360° rotating movements in their head and tail which is not seen in conventional spermatozoa (inlay, circle, see Movie 5). (**e**) Immotile spermatozoa lie flat on the epithelium and lack tail movement (circle, conventional spermatozoona, see Movie 6). (**f**) Hyperactivated spermatozoa are characterised by high amplitude, whip-like tail movements (circle, conventional spermatozoon, see Movie 7). (Scale bars: 10 µm, Inlay 20 µm).

Qualitative digital videomicroscopy within the oviduct was performed in conventional and sexed spermatozoa. Figures and movies were captured within the first 45 min of the formation of the sperm reservoir in time intervals of 15 min. Preliminary studies were performed which revealed that the patterns of sperm movement were not affected by cycle stage. As a consequence, the diestrus cycle stage was selected for the experiments. The overall sperm movement patterns were similar in conventional and sex-sorted spermatozoa. Live cell imaging of the oviductal sperm reservoir of conventional and sex-sorted spermatozoa in real-time showed that most of the spermatozoa in the reservoir were agile spermatozoa. They were characterized by binding to the tubal cilia at a tangential angle of about 30° with an actively beating, undulating tail (Figs. 1a,b, Movies 1 and 2). Agile spermatozoa made up the highest percentage of sperm in the conventional (Fig. 1a, Movie 1) and sexed sperm reservoirs (Fig. 1b, Movie 2). Figures 1a,b and Movie 1 and 2 show a view on the top of the ampullar inner surface. Figure 1c shows a bound spermatozoon from a lateral view highlighting the characteristic binding angle of an agile spermatozoon. The precise movement of an agile spermatozoon (labelled no. 2) is shown in slow motion in Movie 3.

Within the sperm reservoir a small number of spermatozoa revealed a decreased binding angle with a slightly moving head and reduced motility of the tail which were referred to as lagging spermatozoa (Fig. 1d, Movie 4). This pattern of sperm binding was seen both in conventional and sex-sorted spermatozoa. However, in individual sex-sorted spermatozoa (seen in 3 different bulls) a modified pattern of sperm behaviour was observed, which was associated with a 360° rotational movement of the head and tail. This behaviour was never seen in conventional sperm (Fig. 1d, Inlay, Movie 5).

In both conventional and sex-sorted sperm reservoirs, individual spermatozoa were lying flat on the epithelium with an immotile tail. Spermatozoa which were immotile before attaching to the epithelium were flushed away due to the current produced by the ciliary beating. A percentage of spermatozoa became immotile after binding to the tubal epithelium resulting in the amount of immotile sperm continuously increasing over time. Importantly, the sex-sorted spermatozoa revealing the distinct rotational movement of the head and tail were stopping their tail beat and were immotile within a few minutes. Immotile spermatozoa were lying flat and were stuck to the epithelium (Fig. 1e, Movie 3, no. 1, Movie 6). In each sperm reservoir there were single sperm revealing hyperactivation, which was characterized by an asymmetrical, high amplitude and whip-like beating of the tail and a moving head (Fig. 1f, Movie 3, no. 3, Movie 7). To provide a comprehensive overview and comparison of the different patterns sperm movement within the tubal reservoir, Movie 3 shows in slow motion agile (2), immotile (1) and hyperactivated (3) spermatozoa located beside each other in the sperm reservoir from a lateral view.

Overall, immotile spermatozoa, which were present after freezing and thawing of conventional and sex-sorted spermatozoa, were not able to bind and were flushed away by the current produced by ciliary beating. Further to that, a part of the motile spermatozoa were not able to bind. Spermatozoa, which were not able to bind to the oviductal cilia, became immotile within a few minutes and were also flushed away by the fluid flow generated by ciliary beating.

**Figure 2:**
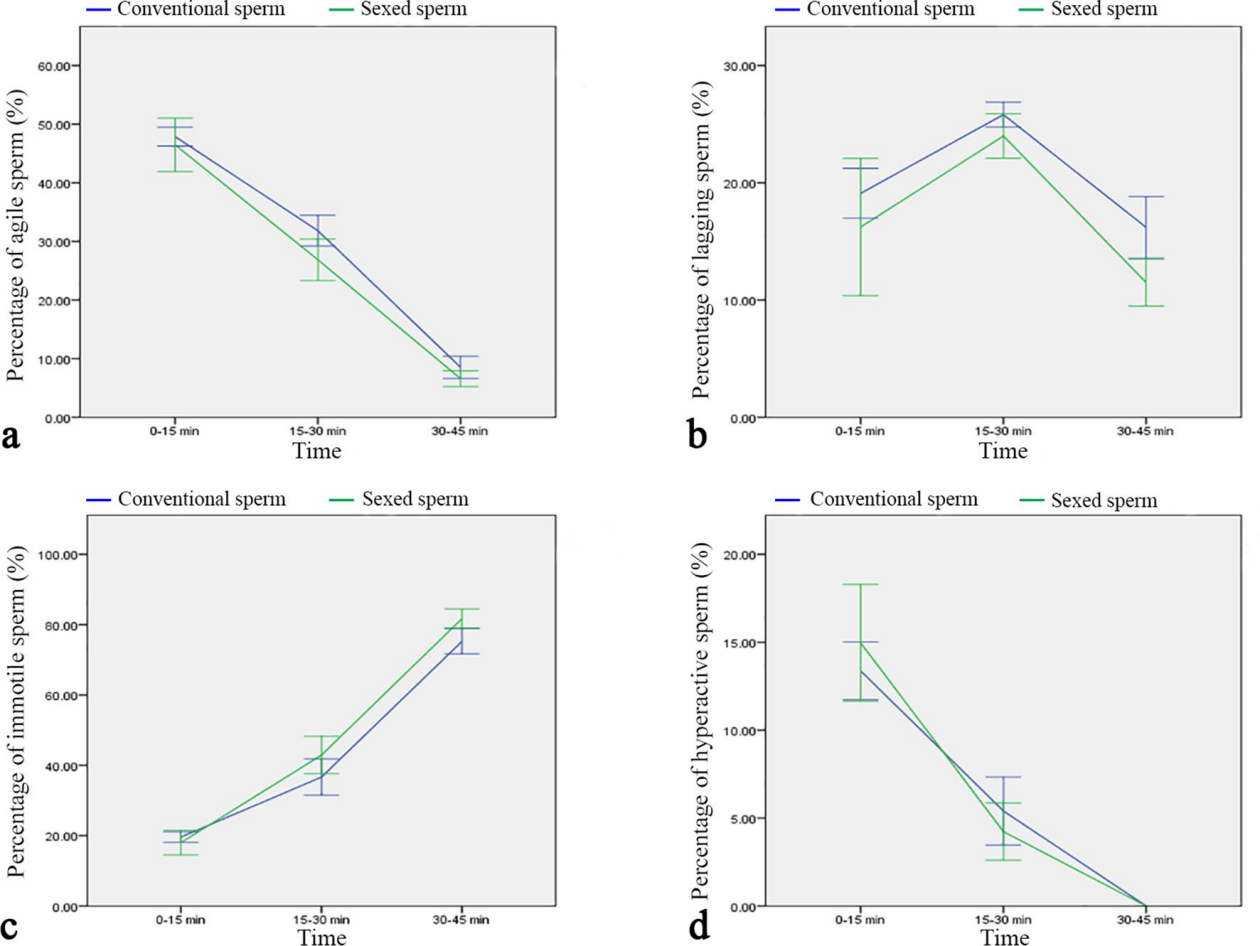
Time-related quantitative analysis of conventional and sexed bovine sperm behaviour within the sperm reservoir (n = 7 bulls, n = 14 ampullae). (**a**) Agile spermatozoa are most prevalent immediately after formation of the sperm reservoir and continuously decrease over time. There is no difference in the prevalence of agile spermatozoa between conventional and sexed sperm reservoirs (Kolmogorov–Smirnov test for normality, area under the curve, *p* = 0.324). (**b**) 10–20% of spermatozoa bound in the reservoir are lagging spermatozoa. The prevalence of lagging spermatozoa within the reservoir is similar in conventional and sex-sorted spermatozoa (Kolmogorov–Smirnov test for normality, area under the curve, *p* = 0.427). (**c**) Immotile spermatozoa are most prevalent 30–45 min after formation of the sperm reservoir. The prevalence of immotile spermatozoa in the reservoir does not differ between conventional and sexed spermatozoa (Kolmogorov–Smirnov test for normality, area under the curve, *p* = 0.185). (**d**) 5–15% of the spermatozoa in the reservoir are hyperactivated spermatozoa, the number of which decreases over time. The prevalence of hyperactivated spermatozoa is similar in conventional and sexed sperm reservoirs (Kolmogorov–Smirnov test for normality, area under the curve, *p* = 0.728). Error bars: + / − 1 standard error mean.

Quantitative live cell imaging studies showed that in bovine frozen-thawed spermatozoa around 50% of total bound spermatozoa had the capacity to bind to the cilia of the ampulla in a tangential angle of about 30° with an actively beating, undulating tail (Fig. 2a). The number of agile spermatozoa in the sperm reservoir continuously decreased with time (Fig. 2a). About 10–20% of the spermatozoa in the oviduct were lagging spermatozoa binding in a reduced angle to the epithelium with a slightly moving tail (Fig. 2b). With progress of time, some agile spermatozoa started to decrease their binding angle and showed reduced tail movement showing the behaviour of lagging spermatozoa. Therefore the percentage of lagging spermatozoa increased over time and was highest after 15–30 min (Fig. 2b). The prevalence of lagging spermatozoa then decreased with time as spermatozoa increasingly became immotile (Fig. 2b). Immediately after formation of the sperm reservoir, 15–20% of bound spermatozoa were immotile (Fig. 2c). The abundance of immotile spermatozoa steadily increased with time (Fig. 2c). Hyperactivation within the sperm reservoir was seen in 5–15% of sperm. If hyperactivation occurred within the sperm reservoir and the spermatozoa maintained binding, the spermatozoa became immotile within 45–60 min resulting in a continuous decrease of hyperactivated spermatozoa with time (Fig. 2d).

Statistical analyses of sperm behaviour of conventional and sexed spermatozoa from the same bulls revealed that sorting did not significantly affect the mean percentage of agile spermatozoa (Fig. 2a), lagging spermatozoa (Fig. 2b), immotile spermatozoa (Fig. 2c) or hyperactivated spermatozoa (Fig. 2d) at all observed time intervals (Kolmogorov–Smirnov test for normality and comparison of the Area under the Curves (AUC), vital sperm: *p* = 0.324; lagging sperm: *p* = 0.428; immotile sperm: *p* = 0.185; hyperactivated sperm: *p* = 0.735). However, in regard to agile sperm the effective size, which represents the standardized mean difference between conventional and sex-sorted spermatozoa, and is calculated by subtraction of the means and division by the standard deviation (SD) of the respective sample population, was 11.04% lower in sexed sperm as compared to conventional sperm. Cohen`s d, which in this case is an indication of the relative difference in sperm behavior, was 0.55, indicating a moderate effect of sorting on the agility of bovine sperm.

### Sperm binding capacity in the tubal reservoir is different in individual bulls and is reduced following sex sorting

**Figure 3:**
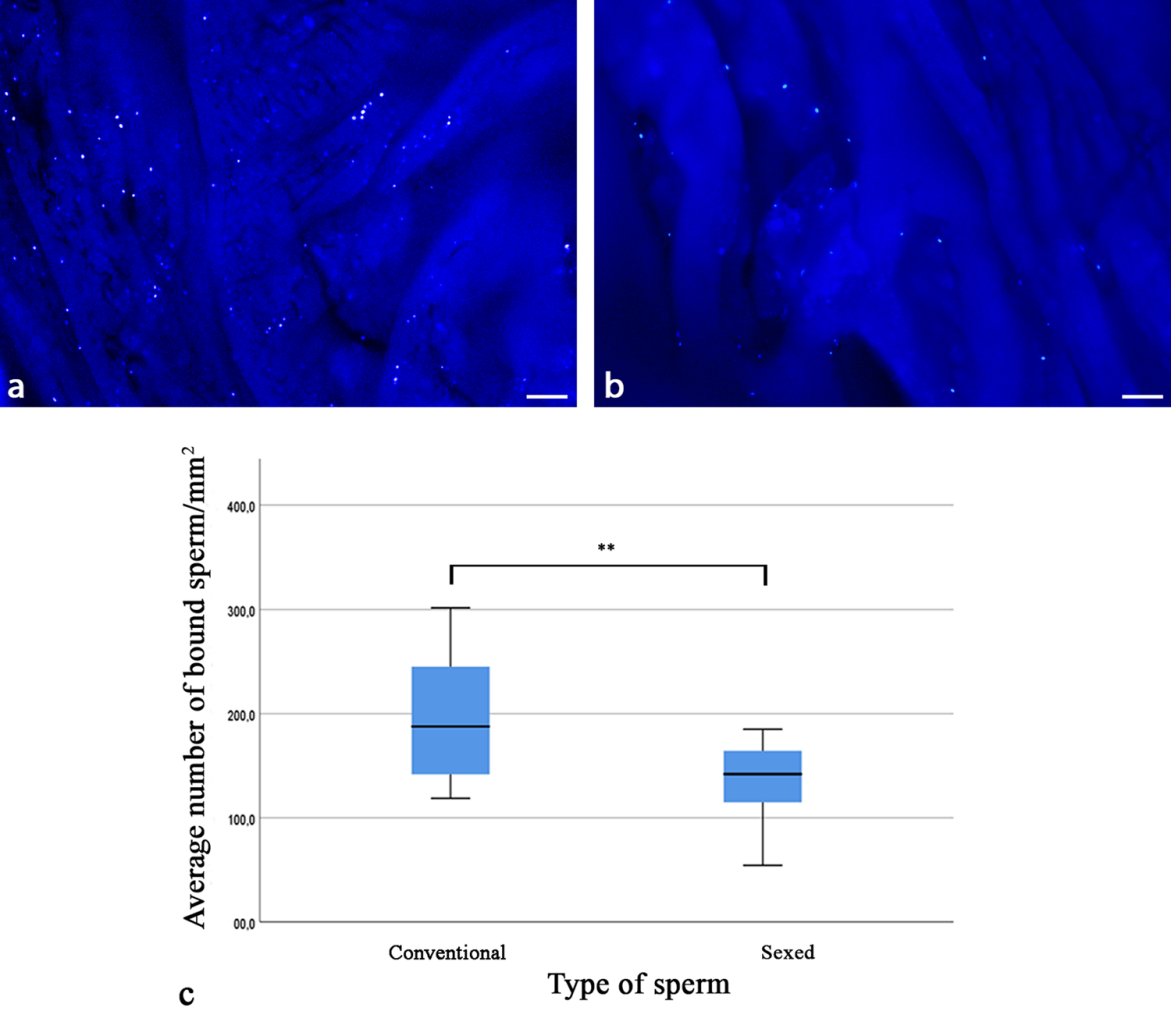
Sperm binding in the tubal sperm reservoir (n = 7 bulls, n = 14 ampullae). (**a-b**) Fluorescent labelling of conventional (**a**) and sexed spermatozoa (**b**) following co-incubation with the oviduct (blue dots). (**c**) Sperm binding capacity is significantly reduced by sex sorting (two-sided paired t-test, *p* = 0.0080). The length of the box represents the interquartile range (IQR), which is the difference between the 75th and 25th percentiles. The line within the box reveals the median.

The number of bound spermatozoa in the oviductal sperm reservoir was analyzed using a newly established fluorescent sperm binding assay (Fig. 3a: conventional spermatozoa, Fig. 3b: sexed spermatozoa of the same bull). This was necessary as identification of native spermatozoa in the oviduct was not completely reliable due to the 3D arrangement of folds and the difficulty to comprehensively localize all spermatozoa in a limited time frame. Sperm concentrations of conventional and sexed spermatozoa were adjusted before starting the experiment. Statistical analyses revealed that significantly less spermatozoa were bound to the tubal epithelium after sex-sorting (Fig. 3c, two-sided paired t-test, *p* = 0.008). The mean number of bound spermatozoa was 197 + / − 69/mm^2^ in conventional spermatozoa, which was reduced to 134 + / − 47/mm^2^ after sex sorting. The relative binding capacity was decreased by 31% + / − 18% in sexed spermatozoa as compared to conventional spermatozoa. Taking the whole oviduct sample into account, on average 0.99% of the conventional spermatozoa and 0.67% of sex-sorted spermatozoa added to the oviduct were bound in the sperm reservoir. There was a distinct individual difference in sperm binding capacity between the bulls, indicated by a coefficient of variation of 35%. After sex sorting, the coefficient of variation between all bulls increased to 58%, indicating that the decrease of sperm binding capacity after sex sorting was even more different and highly individual.

### Oviductal ciliary beat frequency (CBF) is increased after sperm binding: the timely pattern changes after sex sorting

**Figure 4:**
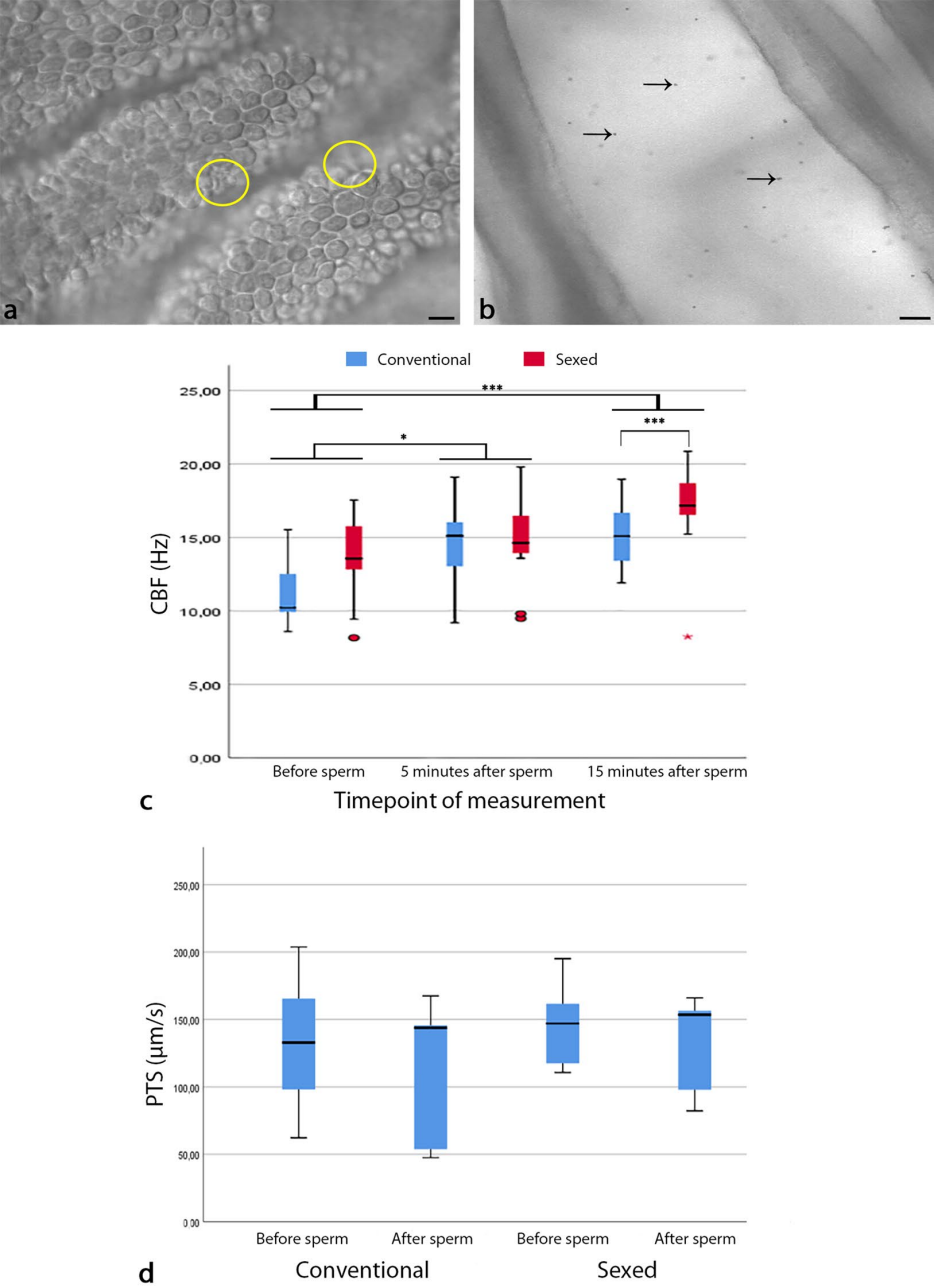
Effects of sperm binding and sex sorting on ciliary beat frequency (CBF, n = 7 bulls, n = 14 ampullae) and particle transport speed (PTS, n = 5 bulls, n = 15 ampullae) (**a**) CBF is calculated by measuring changes in grey scale brightness in ciliated epithelial cells (circles, see Movie 8, part 1). (**b**) PTS is calculated by tracking the path of polystyrene dynabeads (arrows) between oviductal folds (see Movie 8, part 2). (**c**) The addition of spermatozoa significantly increases CBF in the oviduct (generalised estimating equations procedure; before spermatozoa vs 5 min after spermatozoa *p* = 0.022; before spermatozoa vs 15 min after spermatozoa, *p* < 0.001). In sex-sorted spermatozoa CBF is significantly increased after 15 min of co-incubation with the oviduct as compared to conventional spermatozoa (generalised estimating equations procedure; conventional vs sexed spermatozoa; before spermatozoa *p* = 0.732, 5 min after spermatozoa *p* = 0.762, 15 min after spermatozoa *p* < 0.001). (**d**) PTS is not significantly affected by the addition of spermatozoa or by sperm sexing (two-sided Wilcoxon-test for paired samples, conventional spermatozoa *p* = 0.080, sexed spermatozoa *p* = 0.138). (Scale bars: (**a**) 10 µm, (**b**) 20 µm). The length of the box represents the interquartile range (IQR) which is the difference between the 75th and 25th percentiles. The line reveals the median. Circles indicate outliers (values between 1.5 IQR’s and 3 IQR’s from the end of the box) and stars indicate extreme values (values more than 3 IQR’s from the end of the box).

Quantitative live cell imaging revealed that CBF was significantly increased 5 min after the addition of spermatozoa as well as after 15 min (Generalized Estimating Equations procedure (GEE), before sperm versus 5 min after spermatozoa: *p* = 0.022; before sperm versus 15 min after spermatozoa: *p* < 0.001, Figs. 4a,c, Movie 8, Part 1). When comparing conventional and sex-sorted spermatozoa, there was a significant further increase in CBF in sexed spermatozoa after 15 min which did not occur in conventional spermatozoa (GEE, conventional vs sexed spermatozoa: before sperm *p* = 0.732, 5 min after spermatozoa: *p* = 0.762, 15 min after spermatozoa: *p* < 0.001, Fig. 4c).

Further to that, the effect of increased ciliary beating on overall particle transport speed (PTS) was investigated (Fig. 4b, Movie 8, Part 2). PTS is an important indicator for the transport speed of the oocyte after ovulation and the transport of the early embryo^2,61,62^. It is a result of CBF, tubal fluid flow and smooth muscle contraction. Comparison of PTS in the ampulla before and after addition of spermatozoa revealed that PTS was similar before and after the addition of conventional and sex-sorted spermatozoa (Two-sided Wilcoxon-test for paired samples, conventional spermatozoa: *p* = 0.080, sexed spermatozoa: *p* = 0.138, Fig. 4d).

### The presence of spermatozoa and sex sorting do not affect the synthesis of oviductal glycoproteins and acidic mucopolysaccharides

**Figure 5:**
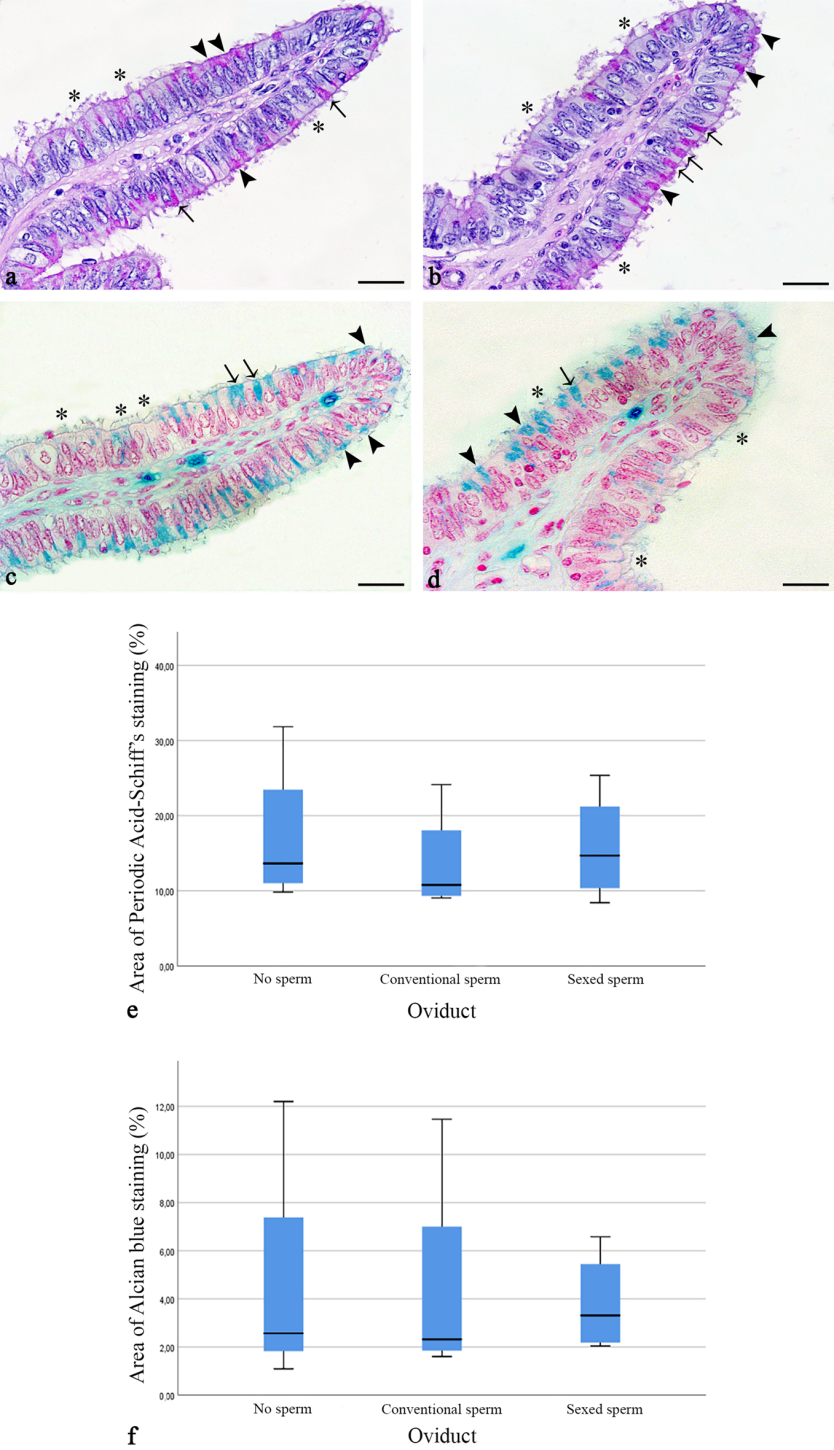
Synthesis of glycoproteins and acidic mucopolysaccharides in the ampulla before and after conventional and sexed sperm binding (n = 4 bulls, n = 12 ampullae) (**a**–**b**) Glycoproteins (pink staining) are localised mainly to the apical cytoplasm of secretory cells (arrowheads) and peg cells (arrows) both in conventional (**a**) and sexed sperm (**b**). Asterisks mark ciliated cells. (**c**–**d**) Acidic mucopolysaccharides are localised to the cytoplasm of secretory cells (arrowheads) both in conventional (**c**) or sexed spermatozoa (**d**). Asterisks mark ciliated cells. (**e**) The synthesis of ampullar glycoproteins is not affected by sperm binding (related-samples, Friedman's two-way analysis of variance by ranks, *p* = 0.135) or sex sorting (Wilcoxon signed ranks test, *p* = 0.655). (**f**) Similarly the expression of oviductal acidic mucopolysaccharides is not affected by sperm binding (related-samples, Friedman's two-way analysis of variance by ranks, *p* = 0.472) or sex sorting (Wilcoxon signed ranks test, *p* = 0.715). (Scale bars: 20 µm). The length of the box represents the interquartile range (IQR) which is the difference between the 75th and 25th percentiles. The line reveals the median.

In order to determine whether glycoprotein and mucopolysaccharide synthesis in the oviductal ampulla are altered by the binding of spermatozoa and by sex sorting, the Periodic acid-Schiff (PAS) reaction and Alcian blue staining were applied to oviducts before and after addition of conventional and sexed spermatozoa, respectively. Before and after the addition of spermatozoa, glycoproteins were localised to the apical membrane of the ciliated cells (Figs. 5a,b, pink staining, asterisks) as well as the apical cytoplasm of secretory cells (Figs. 5a,b, pink staining, arrowheads) and peg cells—slim, non-ciliated cells following the release of secretions (Figs. 5a,b, pink staining, arrows). As shown by identical localization of the signal after amylase digestion, glycogen was not synthesized during diestrus (Figs. 5a,b). Statistical analysis showed that glycoprotein synthesis was not significantly affected by the addition of spermatozoa (Fig. 5e, related-samples Friedman's two-way analysis of variance by ranks, *p* = 0.135) or by sorting (Fig. 5e, Wilcoxon signed ranks test, *p* = 0.655). Acidic mucopolysaccharides, as visualised with the Alcian blue reaction at pH 2.5, were found mainly in the cytoplasm of secretory cells (Figs. 5c,d, blue staining, secretory cells: arrowheads, ciliated cells: asterisks, peg cells: arrows). Statistical analyses revealed that synthesis of acidic mucopolysaccharides in the sperm reservoir was not significantly affected by the addition of spermatozoa (Fig. 5f, related-samples Friedman's two-way analysis of variance by ranks, *p* = 0.472) or by sorting (Fig. 5f, Wilcoxon signed ranks test, *p* = 0.715).

### Spermatozoa display a characteristic surface morphology within the sperm reservoir which is impacted by sex sorting

**Figure 6:**
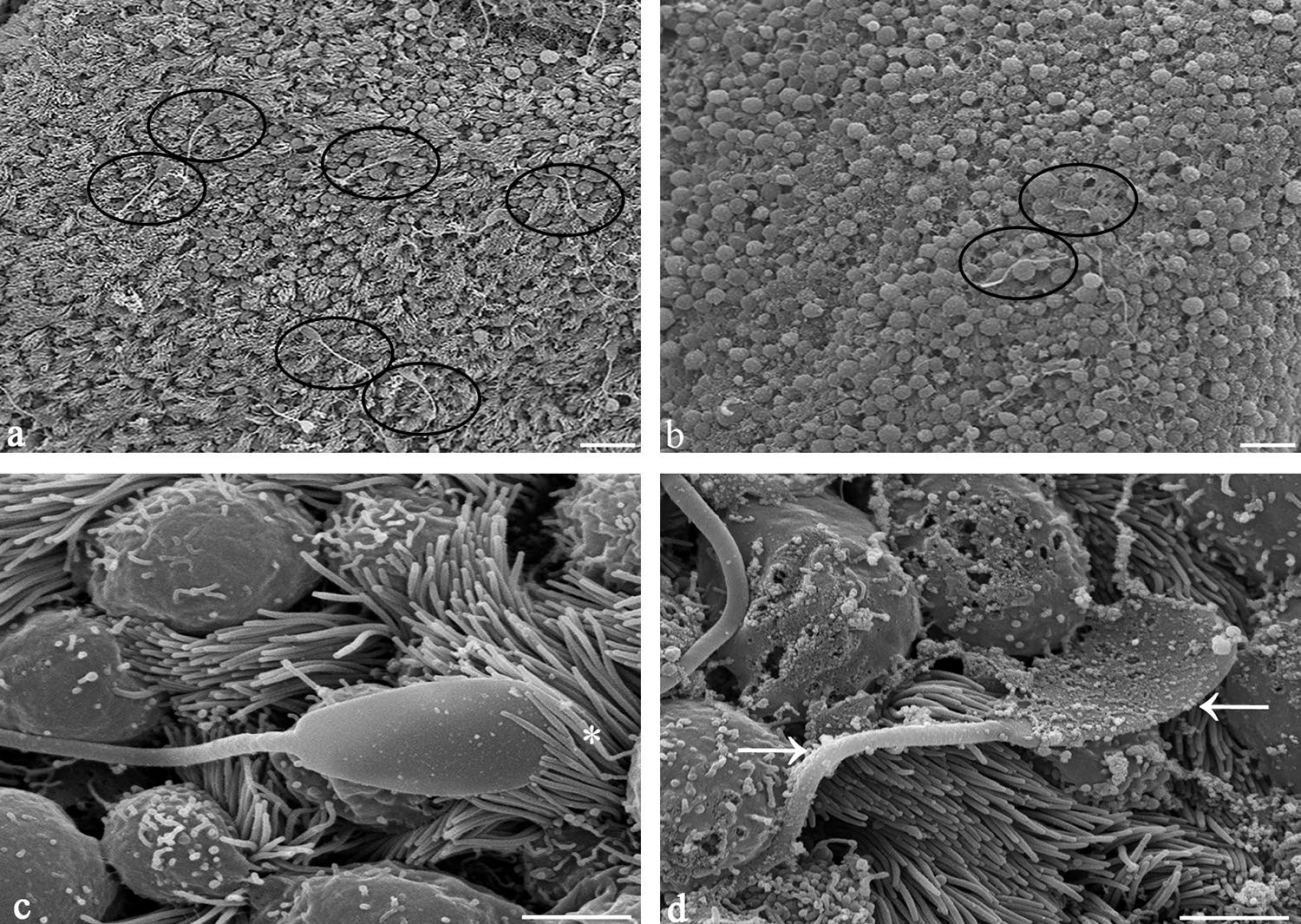
Sperm binding and sperm surface morphology in the sperm reservoir as seen by scanning electron microscopy (n = 5 bulls, n = 15 ampullae). (**a**–**b**) The number of spermatozoa (circles) bound in the reservoir is reduced in sexed sperm (**b**) as compared to conventional sperm (**a**). (**c**) Conventional spermatozoa bind to the ampullar ciliated epithelial cells in a tangential angle and are embraced by the tubal cilia (asterisk). (**d**) After sex sorting a high percentage of spermatozoa reveals deformations in the head and neck and sharp bends in the tail (arrow). The head is covered by secretions and the cilia do not embrace the sperm head. The binding angle is reduced. (Scale bars: (**a**–**b**) 20 µm, (**c**–**d**) 5 µm).

Scanning electron microscopic analyses of the inner surface of the oviduct after co-incubation with conventional and sexed spermatozoa confirmed the results of the sperm binding assay. The number of bound spermatozoa in the sperm reservoir was reduced in sex-sorted spermatozoa (Fig. 6b) as compared to conventional spermatozoa (Fig. 6a). Sperm concentrations of sexed and conventional spermatozoa had been adjusted before the experiments. The majority of conventional spermatozoa within the sperm reservoir revealed a smooth head with intact plasma membrane, a regular neck and midpiece (Fig. 6c). They were bound at a 30° angle and their head was embraced by the tubal cilia (Fig. 6c, asterisk). In contrast, the majority (60–80%, bull-dependent) of sexed spermatozoa revealed deformations in the head and neck as well as sharp bends in their tails (Fig. 6d, arrow). They were bound to the epithelium in a reduced binding angle and were not embraced by the cilia (Fig. 6d). The surface of the head was covered with secretions. However, importantly, there were also a number of sex-sorted spermatozoa bound in the reservoir that revealed binding and sperm morphology resembling conventional spermatozoa.

### Spermatozoa in the tubal reservoir reveal a specific ultrastructure which is significantly altered after sex sorting

**Figure 7:**
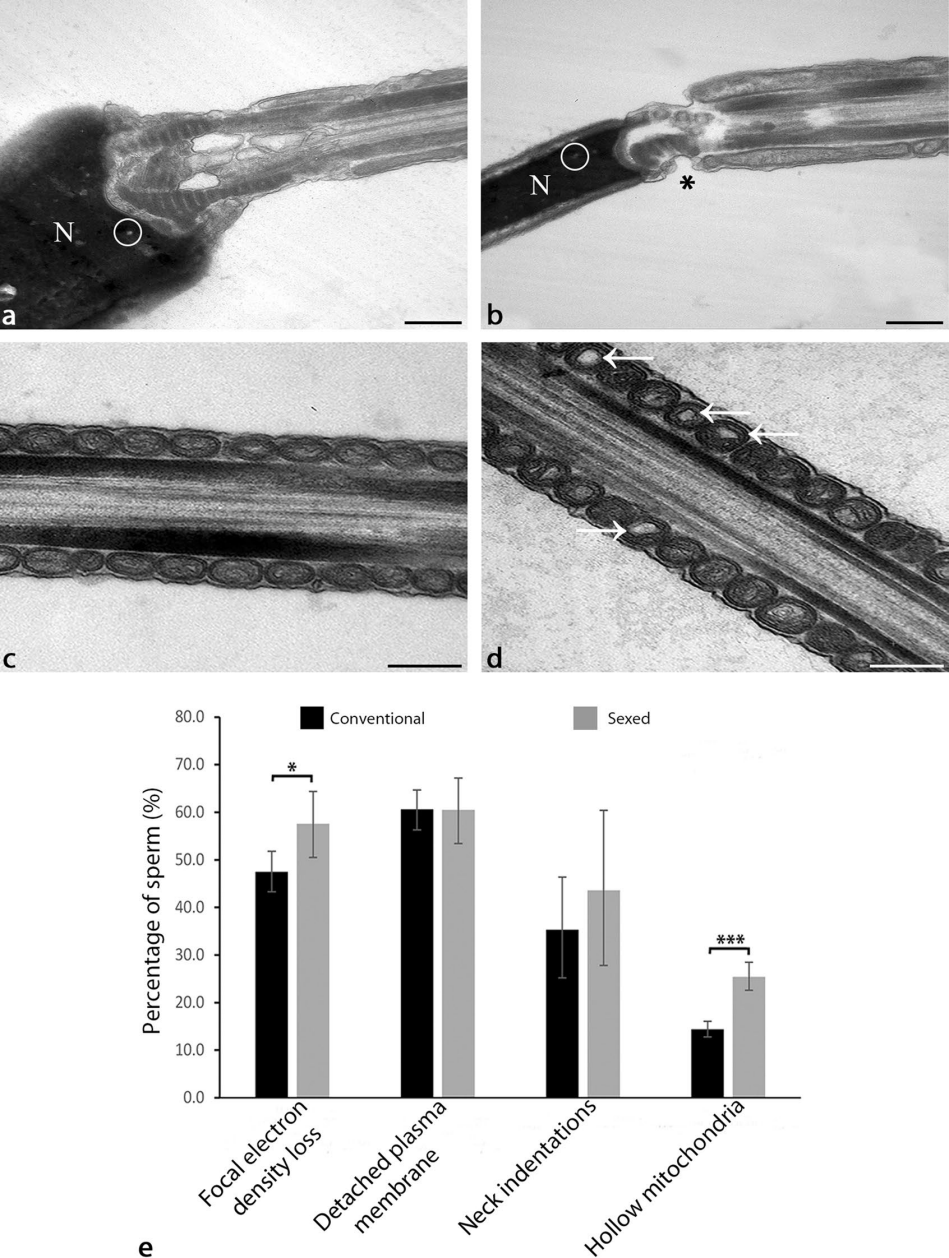
Ultrastructure of spermatozoa within the sperm reservoir as seen by transmission electron microscopy (n = 3 bulls, n = 750 spermatozoa, n = 6 ampullae). (**a**) Spermatozoa reveal a focal loss of electron density in the nucleus (conventional spermatozoa, circle). (**b**) Besides a loss of electron density (circle) sexed spermatozoa also reveal indentations in the neck (asterisk). (**c**) The midpiece in conventional spermatozoa generally reveals mitochondria with regular cristae. (**d**) The midpiece in sexed spermatozoa shows mitochondria with an electron lucent centre and irregular cristae (hollow mitochondria, arrows). (**e**) The focal loss of electron density is significantly higher in sex sorted spermatozoa compared to conventional spermatozoa (Chi Square Test, *p* = 0.014). The prevalence of the detachment of the plasma membrane is similar in sexed and conventional spermatozoa. Indentations of the neck occur in 43.6% of the sexed spermatozoa compared to 35.3% in conventional spermatozoa. The prevalence of hollow mitochondria is significantly increased in sexed spermatozoa compared to conventional spermatozoa (Chi Square Test, *p* < 0.001). (N: nucleus). (Scale bars: 0.25 µm). The error bars indicate the 95% confidence interval.

**Figure 8:**
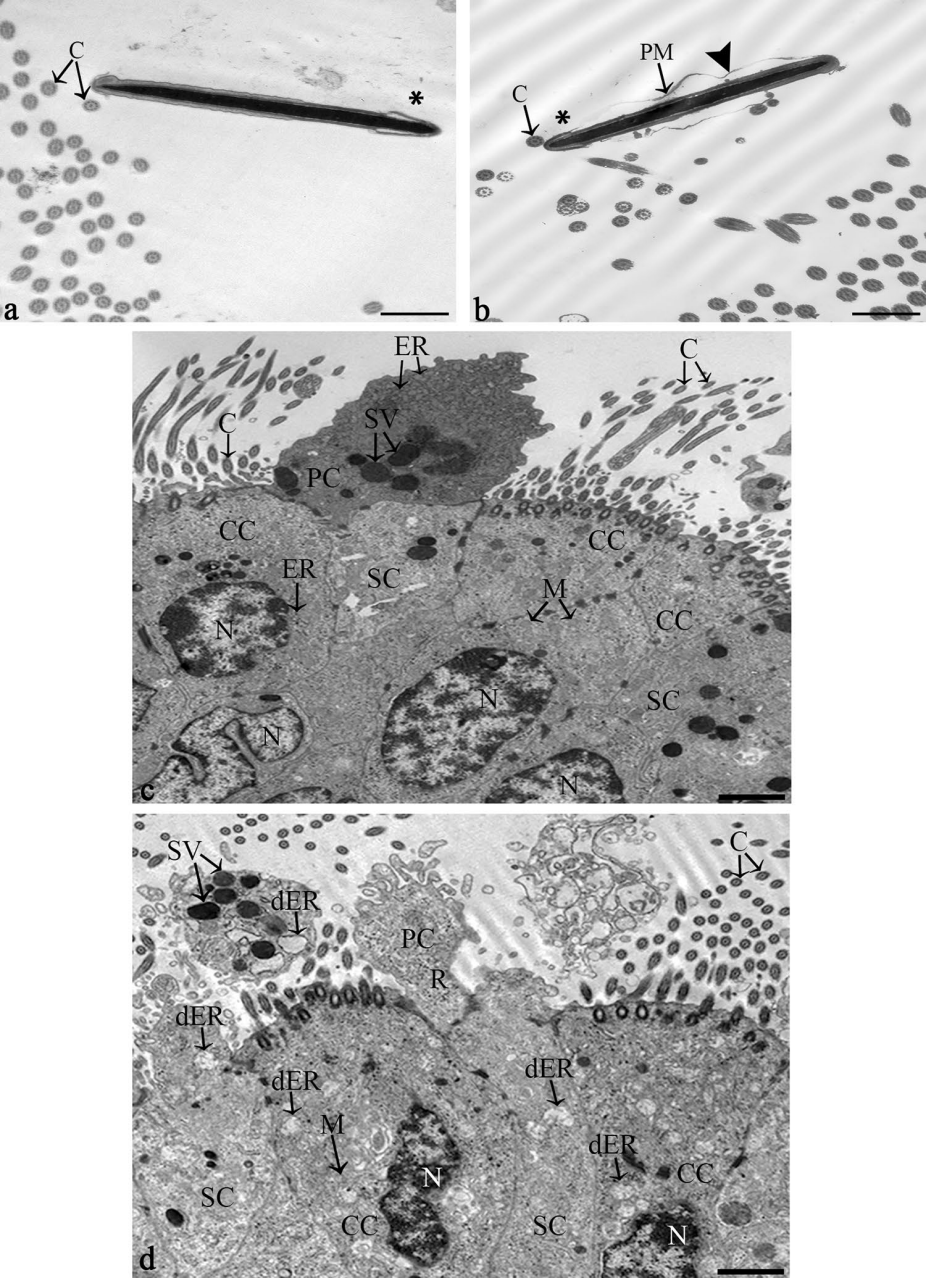
Ultrastructure of the conventional and sexed sperm reservoir and the tubal epithelial cells before and after sperm binding (n = 3 bulls, n = 9 ampullae). (**a**–**b**) Both conventional (**a**) and sexed (**b**) spermatozoa bind to the oviductal cilia. Detached plasma membranes (PM) are seen in conventional and sex-sorted spermatozoa (arrowhead). In sex-sorted sperm the distal part of the head (asterisk) is often seen near cilia (C). (**c**) In the bovine ampulla in mid diestrus the tubal epithelium shows high amounts of secretory vesicles (SV), rough endoplasmic reticulum (ER) lined by ribosomes (R), and mitochondria (M) in the ciliated cells (CC), secretory cells (SC) and protruding cells (PC). After formation of the sperm reservoir predominantly dilated endoplasmic reticulum (dER) is seen in the ciliated cells (CC), secretory cells (SC) and protruding cells (PC). (C: cilia, PM: plasma membrane, ER: endoplasmic reticulum, dER: dilated endoplasmic reticulum, M: mitochondrium, N: nucleus, R: ribosome, SV: secretory vesicles). (Scale bars: (**a**, **b):**0.5 µm, (**c**, **d**):1 µm).

The ultrastructure of the head, neck and midpiece of conventional and sexed spermatozoa in the tubal sperm reservoir as well as the ultrastructure of the epithelial cells after sperm binding (n = 6) was investigated by transmission electron microscopy (TEM). Both in conventional and sexed spermatozoa, a focal loss of electron density indicating a difference in DNA packaging was visible in the nucleus (Fig. 7a: conventional spermatozoon, Fig. 7b: sexed spermatozoon, circle, N: nucleus). Frequently, a detachment of the plasma membrane (PM) of the head was seen (Fig. 8b: sexed spermatozoon, arrowhead). Further to that, indentations of the sperm neck occurred (Fig. 7b: sexed spermatozoon, asterisk). In regards to the midpiece of the sperm tail, sexed spermatozoa in particular revealed high amounts of mitochondria displaying a more than 30% loss of cristae in the centre, which were termed hollow mitochondria (Fig. 7c: conventional spermatozoa, Fig. 7d: sexed spermatozoa, arrows). Statistical analyses revealed that the prevalence of hollow mitochondria was significantly increased in sex-sorted spermatozoa compared to conventional spermatozoa (Fig. 7e, 25.4% vs 14.4%, Chi Square Test, *p* < 0.001). The odds ratio was 2.03, indicating a major effect of sex sorting on mitochondrial integrity. Further to that, the focal loss of electron density was significantly higher in sex-sorted spermatozoa compared to conventional spermatozoa (Fig. 7e, 57.6% vs 47.5%, Chi Square Test, *p* = 0.014). The odds ratio was 1.5. Indentations of the neck occurred in 43.6% of sexed sperm compared to 35.3% in conventional sperm (Fig. 7e, Chi Square Test, *p* = 0.379). The odds ratio was 1.42. The prevalence of the detachment of the plasma membrane was similar in sexed and conventional spermatozoa (Fig. 7e, 60.5% vs 60.6%, Chi Square Test, *p* = 0.988).

When comparing the ultrastructure of spermatozoa bound to the cilia of the ampullar epithelium, it became obvious that conventional spermatozoa primarily bound with the front end of the sperm head (Fig. 8a, asterisk: posterior part of the head). In contrast, sexed spermatozoa were frequently in contact with the posterior part of the head (Fig. 8b: asterisk). This related to the fact seen in SEM that conventional spermatozoa mostly bound in a tangential angle of about 30° with the tip of the head contacting the cilia whereas sexed spermatozoa often revealed a reduced binding angle contacting the epithelium with a larger part of the head.

### Sperm binding in the tubal reservoir induces increased activity of the endoplasmic reticula in tubal cells

For analysing tubal reactions after sperm binding, the bovine ampulla in cows during mid-diestrus was investigated. The reason for analysing this cycle stage was to be able to clearly discriminate between reactions induced by local actions of the sperm and systemic hormonal changes. During mid-diestrus, the tubal epithelium was composed of ciliated cells (CC), secretory cells (SC), protruding cells (PC) and basal cells (Fig. 8c). CCs, SCs and PCs showed high amounts of secretory vesicles (SV), rough endoplasmic reticula (ER) and mitochondria (M) in their cytoplasm. After formation of the sperm reservoir, a major change occurred in the endoplasmic reticula, which were largely dilated in CCs, SCs and PCs (dER) after sperm binding (Fig. 8d). This was not seen in the ampullar epithelial cells in the absence of spermatozoa (Fig. 8c). The dilated rER was seen both after binding of conventional and sex-sorted spermatozoa (Fig. 8d, conventional sperm).

## Discussion

Our study provides the first comprehensive documentation of sperm-oviduct interactions in real-time and under near in vivo conditions using the bovine as a model. Further to that, it highlights for the first time how physical and chemical stress impact the formation of the physiological sperm reservoir and the communication of spermatozoa and tubal epithelium.

The applied “ex vivo” oviduct model is superior to traditional oviduct cell culture systems as it mimics the cellular relationships and the biophysical properties of the oviduct. The whole organ including epithelium, underlying connective tissue and smooth muscle are maintained in their physiological arrangement. This ensures morphological integrity of the epithelial cells and supports ongoing physiological function of epithelial hormone and protein receptors as well as maintenance of epithelial signalling cascades and trafficking of molecules^9,63^. As both the ampulla and the isthmus are involved in formation of the sperm reservoir, both parts can be used as models^1–3,64^. In preliminary studies we found that there are no differences between ampulla and isthmus regarding sperm behaviour and sperm movement patterns. Suarez et al. (2006) reported that after ovulation most sperm in the ampulla were located near the cumulus-oocyte-complex^3^. Consequently, the ampulla was chosen for the experiments as spermatozoa bound in the ampulla are those who are prone to be the first spermatozoa to reach the oocyte after ovulation. The cycle stage diestrus was chosen as preliminary studies had shown that the sperm reservoir was formed independent of the cycle stage and that there were no differences in sperm behaviour and sperm movement patterns in the different cycle stages. More importantly, the cycle stage diestrus was selected to be able to discriminate between systemic hormonal effects during estrus and the local effects on the tubal epithelium induced by sperm binding. Regarding the number of spermatozoa applied to the oviduct, from about one million spermatozoa added, only 1% of conventional and 0.7% of sex-sorted spermatozoa were bound in the sperm reservoir, highlighting the function of the oviduct as a site of sperm selection. The reason for these low percentages of spermatozoa within the sperm reservoir was that immotile spermatozoa were not able to bind and were flushed away by the current of the ciliary beating. Additionally, multiple motile spermatozoa were not able to bind and were flushed away, pointing to a possible damage of receptors on the plasma membrane after freezing and thawing. The number of spermatozoa bound in our model is still higher than the number of spermatozoa physiologically reaching the oviduct^8^. However, in experimental models it is often of advantage if reactions are extensively triggered to be able to identify them. Consequently, if more spermatozoa are bound than in vivo, the reactions induced by sperm binding are more obvious and even minimal reactions, which were not recognized if only single spermatozoa were bound, can be identified.

Our live cell imaging studies revealed that, within the sperm reservoir, agile spermatozoa are the dominant sperm population. They are characterized by a tangential binding angle of around 30° with an actively beating, undulating tail. As shown by SEM, the cilia embrace the tip of the conventional sperm head in a characteristic manner. After physical and chemical stress – as occurring during sex-sorting – this embracement is largely lost, pointing to a lack of recognition by the tubal cilia. This reduced ability of mechanical interaction between spermatozoa and oviductal epithelial cells is reflected in the reduced binding capacity of sex-sorted spermatozoa within the tubal sperm reservoir, which we showed using a novel sperm binding assay performed within the oviduct. These results are confirmed by findings in vitro that sex-sorted sperm binding in bovine tubal explants is diminished after 24 h^31^. In contrast to our studies, the authors did not observe a difference within the first 30 min which might be due to the in vitro conditions, which are associated with partial loss of cell function, and the use of single oviductal cells instead of the whole organ, which mimics the cellular relationships and the biophysical properties of the tubal epithelium^65^.

The observed immediate reduction in sperm binding capacity after sex-sorting might be due to the damage or removal of receptor proteins on the sperm heads by physical strains. Located on the plasma membrane of the head, species-specific lectins mediate the sperm binding to sugars of the oviductal cilia^8,10–12^. In the cow, the carbohydrate moiety on the cilia is fucose, which binds to the sperm head proteins BSP1 (aka PDC-109 or BSP-A1/A2), BSP3 (BSP-A3) and BSP5 (BSP-30 kDa)^13^. Each of these proteins alone can mediate the binding of bovine spermatozoa to the tubal epithelium^19,30,66^. To date, 58 sperm proteins located on the bovine sperm head have been identified^67^. Thus, damage to the head of the spermatozoa might result in altered function of these lectins, resulting in compromised initiation of the calcium signalling cascades which are induced after sperm binding^68^. This is supported by our finding that individual sex-sorted spermatozoa in the reservoir show a 360^o^ rotation of head and tail in our live cell imaging studies – a behavioural pattern that was never observed in conventional spermatozoa and which might be related to the genetic background of the bulls revealing this specific movement of sex-sorted spermatozoa. It has been described that sex-sorted spermatozoa present with a “pre-capacitation” state which includes changes to the plasma membrane which are similar to those seen in capacitated spermatozoa^31^. Supplementation of sex-sorted spermatozoa with seminal plasma from different, highly reproductive quality bulls might support sperm binding capacity after sex-sorting. To apply seminal plasma after sex-sorting might be important as it is known that removal of seminal plasma before the sex-sorting process improves sorting rate, increases sperm motility and reduces damage to sperm DNA and the sperm plasma membrane^69^. Further to that, supplementation of BSP proteins might hold promise to improve sperm binding capacity, as has been documented in capacitated sperm.

In our live cell imaging studies, it was obvious that immotile spermatozoa are not able to bind to the oviductal epithelium and are flushed away by the current of the ciliary beating. The reduced motility documented in sex sorted spermatozoa might contribute to the reduction in sperm binding capacity. Further to that, various motile spermatozoa are also not able to bind, pointing to a possible damage of receptor proteins during freezing and thawing. Spermatozoa which are not able to bind become immotile within a few minutes and are also flushed away by the fluid flow. These findings show that the oviduct is the third major site of sperm selection besides the cervix and the uterotubal junction (UTJ). These results also confirm that a lack of sperm binding ability leads to reduced survival time^30,70^. This is further supported by findings of in vitro studies highlighting that after formation of the sperm reservoir the acrosomes are preserved intact over time, thus preserving sperm fertilizing ability^71^. According to our findings, sperm fertilizing capacity after sex-sorting is further compromised by reduced tail movement, which might partly be due to the morphological sperm alterations seen in SEM and TEM such as indentations in the neck and sharp bends in the midpiece of the tail as well as to the damage of mitochondria observed. Further to that differences in the abundance of sperm head surface molecules in conventional and sexed spermatozoa may also lead to different responses in oviductal epithelial cells following sperm binding. Sexed sperm heads are also covered by increased amounts of secretions, which might be caused by stripping of the BSP proteins exposing surface proteins. As shown in studies of sperm-oviduct interactions in inflamed oviducts, excessive amounts of secretions on the sperm affect the sperm release from the reservoir^61^. Release of sperm from the reservoir is initiated by hyperactivation, which is characterized by asymmetrical flagellar bending with a high amplitude^8^. Hyperactivation also supports the passage of sperm through the tube and enables sperm penetration through the cumulus oophorus of the oocyte^72^. All these events (sperm hyperactivation, detachment from the reservoir, migration and penetration of the cumulus oophorus) may be compromised by accumulation of mucus on the head, as well as by indentations of the neck and sharp bends in the sperm tail (as seen by SEM and TEM) after sex-sorting. The prevalence of hyperactivated spermatozoa has been shown to be significantly decreased in CASA studies after freezing and thawing of sex-sorted bovine spermatozoa^52^. Interestingly, this difference was not the seen in the tubal sperm reservoir after sperm binding to the oviductal cells, highlighting that the tubal cells are able to support sperm motility.

As shown by a coefficient of variation of 35%, the amount of spermatozoa forming the sperm reservoir in the oviduct is highly individual. This means that even spermatozoa from selected ejaculates with excellent morphology, motility and viability will bind and survive in the oviduct in very different numbers. The coefficient of variation rises to 58% after sex-sorting, implying that spermatozoa of some individuals—depending on their genetic background—will cope better with the sex-sorting process than others^73^. As sperm survival and sperm fertilizing capacity can only be maintained within the reservoir, different bulls may be well or badly suited for sex sorting. As the health of the female genital tract plays an additional decisive role for sperm, these individual differences are pivotal for the outcome of assisted reproductive technologies (ART)^61^. Future studies will have to focus on establishing reliable predictors for the stress resistance of spermatozoa and for the performance of spermatozoa after sex-sorting.

As shown by our studies, sperm binding in the oviduct induces a number of tubal reactions, the most conspicuous of all is the increase in ciliary beat frequency (CBF). Similarly, CBF has been shown to be enhanced after sperm binding in rat and human tubal explants *in vitro*^74,75^*.* This increase in CBF might be due to the action of molecules secreted from the oviductal cells after formation of the sperm reservoir^76^. Well known modulators of CBF are progesterone, prostaglandins and adrenomedullin^74,75,77^. Increased CBF after sperm binding might be a valuable physiological tool to increase fluid flow near the cilia in order to enhance the supply of nutrients and to effectively transport signal molecules released after ovulation of the oocyte. Interestingly, after 15 min, the CBF of oviducts incubated with sex-sorted spermatozoa is significantly more increased compared to those with conventional spermatozoa. This tubal reaction might contribute to the removal of the increased number of dead spermatozoa and detritus, thus supporting viable sperm selection and tubal clearance.

Besides CBF, movement of tubal fluid and smooth muscle contraction contribute to gamete transport^2^. Particle transport speed (PTS) is the result of all these factors. As shown by our quantitative live cell imaging studies, PTS is not affected by sperm binding neither in conventional nor in sex-sorted spermatozoa. Similarly, diseases in the bovine female genital tract, such as inflammation, have been shown not to impact PTS^61,76^. These findings point to the fact that the female genital tract tries to maintain a constant transport speed by combining up- and downregulation of CBF, tubal fluid flow or smooth muscle contraction. This is supported by studies in rabbits and rats documenting that the cilia alone are able to transport the gametes and the early embryo within a normal time frame^78,79^. In addition to that, women with Kartagener Syndrome, who have immotile cilia, are able to become pregnant^80,81^. Further to that, as spermatozoa form the sperm reservoir along the inner wall of the oviduct, ciliary activity is prone to have the highest immediate impact on spermatozoa compared to fluid flow and smooth muscle contraction^82–84^.

As indicated by our TEM studies, sperm binding is related with increased activity of the rough endoplasmic reticulum (rER) in the ciliated and secretory tubal cells. This cell organelle plays a pivotal role in protein synthesis and protein trafficking, i.e. the vesicular transport of synthesized proteins (mostly glycoproteins) to the Golgi apparatus^85^. Both conventional and sex-sorted spermatozoa induce the abundant presence of dilated rER in the tubal epithelium. Thus, sperm binding in the reservoir initiates a signalling cascade enhancing the tubal secretory activity and the synthesis of extracellular vesicles (EVs) of the oviduct. This ensures nutrition of the spermatozoa, enables them to undergo capacitation and regulate acrosomal exocytosis and hyperactivation as well as maintain fertilizing capacity^86^. Interestingly, the synthesis of glycoproteins and acidic mucopolysaccharides after sperm binding, as seen by histochemistry, was not increased. This might be due to the fact that the systemic synthesis of glycoproteins in the female genital tract is upregulated around ovulation, which guarantees the presence of these essential molecules when the spermatozoa arrive in the oviduct^22^. Further to that, the tubal cells, after sperm binding, locally produce increased amounts of other molecules such as glycolipids, lipoproteins, enzymes or growth factors^87^.

When analysing the ultrastructure of frozen thawed conventional and sex-sorted spermatozoa, it became obvious that freezing and thawing leads to structural changes of the spermatozoa such as detachment of the plasma membrane, focal density loss in the nucleus and indentations of the neck. These alterations, which occur after freezing and thawing as well as after vitrification of spermatozoa, have been described in canine sperm recently^88^. However, after sex-sorting, the percentage of spermatozoa revealing focal density loss in the nucleus was significantly increased. The focal loss of electron density indicates reduced chromatin condensation, which in spermatozoa is commonly caused by excess production of reactive oxygen species (ROS)^89^. In addition, Balao da Silva et al. (2016) found a simultaneous increase in DNA fragmentation and production of ROS in stallion spermatozoa after sex sorting^53^.

The most striking ultrastructural alteration in spermatozoa after sex-sorting was the significantly enhanced presence of mitochondria in the midpiece which were lacking cristae in their centres (hollow mitochondria). This kind of mitochondrial alteration has been described recently in a mouse model for the human disease chorea-acanthocytosis (ChAc), which is a mutation in the Vacuolar Protein Sorting 13 Homolog A (VPS13A) gene encoding chorein, and which is associated with reduced sperm motility and with infertility^90^. Sperm mitochondria are involved in numerous functions such as ATP production, calcium homeostasis and the intrinsic apoptotic pathway related to the negative effects of sperm sex sorting on sperm motility^91^. Our results of ultrastructural mitochondrial changes are supported by the findings that mitochondria of sex-sorted spermatozoa show reduced membrane potentials and that ATP production in bovine spermatozoa is reduced following sex-sorting^31,92^. These alterations might also critically harm the maneuverability of the sperm tail, which is supported by our findings of abnormal movement patterns of sex-sorted spermatozoa in live cell imaging studies. The impact of mitochondrial altered function might be enhanced by mechanical lesions such as indentations of the neck including the damage of the striated columns and SUN-domain proteins anchoring the head to the tail as well as by sharp bends in the tail as seen by SEM^93^. All over, the morphological alterations observed might play a major role in the reduced fertility of spermatozoa observed after sex-sorting. In the 823 field studies performed to date using sex-sorted semen, the fertility is reduced by 15–18% in heifers and up to 25% in cows^94^. During in vitro embryo production, 4-cell embryos derived from sex-sorted semen reveal increased incidence of arrest, increased shrinkage and fusion of blastomeres. Overall, reduced embryo survival times and decreased blastocyst rates occur, which result in lower numbers of expanded and hatched blastocysts^52^.

In summary, our study is the first to comprehensively document the time-dependent patterns of sperm behaviour within the sperm reservoir in an ex vivo organ model and in real-time using a novel live cell imaging technology. It analyses the tubal reactions occurring after sperm binding and relates them to morphological and ultrastructural changes. The analysis of sex-sorted spermatozoa, which was included in the study, provides a valuable model for the action of mechanical, chemical and time-related stress on sperm morphology and function. Immediate application of our results might create the basis for further improvement of the sex-sorting process, allowing a widespread use of this promising technology in husbandry. Regarding in vitro fertilization (IVF) or intracytoplasmatic sperm injection (ICSI), spermatozoa undergo mechanical stress such as centrifugation after dilution and washing as well as chemical stress by being placed in swim-up media or alternative chemicals used for selection of viable spermatozoa. Thus, the knowledge in which ways mechanical and chemical stress affect sperm morphology and function could help to develop strategies to mitigate these effects, e.g. by implementing an alternative technical procedure or by addition of specific protective molecules. Consequently, looking further, the knowledge of physiological sperm behaviour within the female genital tract and the knowledge of the impact of mechanical and chemical stress on sperm function is pivotal for creating new therapeutic tools for treatment of human subfertility and infertility, especially for further improving the success rates of assisted reproductive technologies (ART).

## Materials and methods

### Ethical approval

This study was ethically approved by the Animal Research Ethics committee of UCD (Koelle AREC-E-18–42). Exemption from full ethical review was granted on the grounds that bovine female genital tracts were obtained from the abattoir after slaughter. All methods were conducted under the guidelines and regulations of the University College Dublin Office of Research Ethics.

### Preparation of bovine spermatozoa

Frozen conventional and sexed spermatozoa from the same bulls (n = 7) were obtained from Rinderzucht Schleswig Holstein eG, RSH, Germany. Conventional and sex-sorted spermatozoa were obtained from the same bulls, but from different ejaculates which had been obtained in similar timeframes. Spermatozoa were sex-sorted using the routinely used flow cytometric sperm sexing technology, which was developed by Johnson et al. in the United States Department of Agriculture named Beltsville Sperm Sexing Technology. It was patented in 1991, and licensed by the USDA to XY Inc, which was acquired by Sexing Technologies (Navasota, TX, USA) in 2007^46,95^.

Conventional and sexed semen straws were thawed at 39 °C for 10 s and transferred to 1.5 ml Eppendorf tubes which were kept at 37 °C. Sperm motility was assessed after thawing and washing on warmed glass slides in 2 biological replicates by the same operator using an Olympus CKX41SF phase contrast inverted microscope (Olympus, Germany). Conventional frozen-thawed spermatozoa had an average post-thaw motility of 79 + /- 5.8% while sex-sorted frozen-thawed spermatozoa had an average motility of 56 ± 11.7%. The spermatozoa were washed with HEPES buffer (5.6 mM KCl, 136.4 mM NaCl, 1 mM MgCl_2_–6H_2_O, 2.2 mM CaCl_2_–2H_2_O, 11 mM glucose and 10 mM HEPES, pH 7.4) and centrifuged at 200 rcf for 2 min. The sperm concentrations of conventional and sexed semen were adjusted after calculating the sperm concentration using a hemocytometer and diluting conventional spermatozoa with HEPES accordingly.

### Collection of bovine genital tracts

Female reproductive tracts were collected from an abattoir (Kildare Chilling Company, Kildare, Ireland) immediately after slaughter and were transported to the lab at 4 °C. All bovine tracts used in the experiments were from cows in all stages of diestrus (corpus luteum in formation, bloom or regression). Only healthy genital tracts were used for the experiments. The cycle stage was determined by gross anatomical inspection (presence of closed cervix, moderate to flaccid muscle tone, lack of secretions and the presence of ovary with a corpus luteum). When comparing the behaviour of conventional and sexed spermatozoa of the same bull we used consecutive pieces of the ampulla of the same cow for comparison to exclude the effects of female variability. The oviducts were dissected from the surrounding mesosalpinx and placed in HEPES buffer (4 °C) for 10 min before the following investigations.

### Qualitative Live Cell Imaging

The ampullae of oviducts (n = 14) were cut into 1 cm pieces, which were opened and pinned onto Delta-T dishes that had been coated with a 2 mm layer of Sylgard gel (Dow Corning, MI, USA). In the following experiments, the ampullae were co-incubated with 1 million (30 μl) conventional or sexed spermatozoa from the same bulls (n = 7) in HEPES buffer (3 ml) at 37 °C for 15 min. The Delta-T dish was then placed onto a BX51 WI fixed-stage upright microscope (Olympus, Hamburg, Germany) with a specific Delta-T dish holder coupled to a stage and objective heater (Bioptechs Inc., Philadelphia, USA). A constant temperature of 37 °C was maintained throughout the experiment. Spermatozoa were classified as either agile (binding at tangential angle of about 30° with an actively beating, undulating tail movement), lagging (binding at lower angle, reduced tail movement at low frequency), immotile (lying flat on cilia, lack of tail movement) or hyperactivated (binding at tangential angle, rotation of the head, whip-like tail movements). For both qualitative and quantitative live cell imaging experiments, a BX51 WI fixed-stage upright microscope (Olympus, Hamburg, Germany) and a 40 × water immersion objective (UMPLFL 40 × W/0.8, Olympus, Hamburg, Germany) were used for analysis and documentation. About 300 spermatozoa were investigated in each biological replicate. For recording of the videos of sperm binding to the oviduct in time intervals of 0, 15, 30, and 45 min the camera Mx7 (1280 × 1024 pixel chip, Sumix, CA, USA) and the software StreamPix 7.0 (NorPix, Canada) were applied.

### Quantitative live cell imaging: analysis of ciliary beat frequency (CBF)

Ciliary beat frequencies in the tubal ampulla (n = 14) before and after contact with spermatozoa and after addition of conventional and sexed spermatozoa of the same bulls (n = 7) were compared. Spermatozoa and oviducts were co-incubated as described before. Ciliary beat frequency (CBF) was determined at three timepoints: before the addition of spermatozoa, immediately following the addition of spermatozoa and 15 min thereafter. At each timepoint, five regions of interest were selected and recorded using a 40 × water immersion objective (UMPLFL 40 × W/0.8, Olympus, Hamburg, Germany). Videos were recorded using Streampix 7.0 (NorPix, Canada) (100 frames per second, 15 s). CBF was calculated as changes to the grey-scale brightness due to ciliary beating using ImagePro (MediaCybernetics, PA, USA). This was then converted to a frequency using Fast Fourier Transformation (FFT) using the software Auto Signal (Systat, California, USA)^63,76^.

### Quantitative live cell imaging: analysis of particle transport speed (PTS)

Particle transport speed (PTS) was analysed in the ampulla (n = 15) before and after contact with spermatozoa and after addition of conventional and sexed spermatozoa of the same bulls (n = 5). For this purpose, spermatozoa were co-incubated with oviducts as described before. 3 µL of dynabeads with a diameter of 2.8 µm (3 × 10^6^/μl Life Technologies, Norway) were added to the HEPES buffer before or after sperm co-incubation. To keep the particles from settling, the buffer was gently mixed before every recording and left to settle for 1 min. A region of interest (ROI, 0.07 mm^2^) was deemed suitable if two oviduct folds were in view and in focus. Particle transport speed (PTS) was recorded in 3 planes of view per ROI (120 frames per second, 15 s) and the dynabeads were automatically tracked using a 20 × water immersion objective (UMPLFL 20 × W/0.8, Olympus, Hamburg, Germany). Analysis was performed using the software ImagePro (MediaCybernetics, PA, USA). Only dynabeads that could be tracked for at least 10 frames were analysed.

### Fluorescent sperm binding assay

In order to compare conventional and sexed sperm binding capacity in the oviduct, a novel fluorescent sperm binding assay including fixation had to be established. This was necessary as counts of native bound spermatozoa in the oviduct using phase contrast microscopy turned out not to be sufficiently reliable due to the complex 3D structure of the ampullar folds. For the binding assay, conventional and sexed spermatozoa from the same bulls (n = 7, n = 14 ampullae) were thawed and washed in HEPES buffer. Sperm concentrations were adjusted as described before. Conventional spermatozoa were stained with the DNA binding dye Hoechst 33,342 (20 µM) for 5 min at 37 °C. Sexed spermatozoa were already stained with Hoechst 33,342 during the sorting procedure. Staining efficiency of spermatozoa was determined by merging phase contrast microscopic and fluorescent images of 5 regions of interest (ROIs) in a 7 μl drop of sperm dried on a coated slide at 37 °C for 5 min using the software Adope Photoshop CC 2018. Only samples with a staining efficiency of > 70% were included in the study. Following staining, spermatozoa were co-incubated with 1 cm pieces of ampulla (n = 14) for 10 min at 37 °C. After fixation in a 4% glutaraldehyde solution in Sorenson’s buffer (1:5 (v/v) solution of 0.07 M KH_2_PO_4_ and 0.07 M Na_2_HPO_4_–2H_2_O) at 4 °C for 5 min, the oviducts were cut open longitudinally and placed onto Superfrost Plus slides (Thermo Scientific, Dublin, Ireland) with the mucosa facing upwards. Bound fluorescent spermatozoa were documented and counted in 5 ROIs using an Olympus BX51 microscope (Olympus, Hamburg, Germany) and a DP71 camera (Masontec, Dublin, Ireland). On average, 166 spermatozoa/mm^2^ were counted. For statistical analysis, the staining efficiency was implemented in the final calculations.

### Histochemical analyses of the synthesis of glycoproteins and acidic mucopolysaccharides

For investigating the oviductal synthetic activity of glycoproteins and acidic mucopolysaccharides after sperm binding, 1 cm samples of ampulla (n = 12) were incubated with (a) HEPES buffer—no spermatozoa, (b) conventional spermatozoa and (c) sexed spermatozoa from the same bulls (n = 4) for 10 min at RT. Tissues were fixed in Bouin’s solution for 24 h and dehydrated in ascending series of ethanols (70–100%). After embedding in paraffin, Sects. (5 µm) were cut and mounted onto Superfrost Plus slides (Thermo Scientific, Dublin, Ireland). After rehydration of the sections in descending series of ethanols (100%, 100%, 80%, 70%, 5 min each), glycogen and glycoproteins in the oviduct were localised using the Periodic Acid Schiff (PAS) reaction. The production of acidic mucopolysaccharides in the oviducts before and after contact with conventional and sexed spermatozoa was investigated using Alcian blue staining pH 2.5. Three technical replicates of each staining procedure were performed.

For PAS staining, rehydrated sections were placed in 0.5% periodic acid (Sigma-Aldrich, Wicklow, Ireland; 5 min), followed by running water (10 min), Schiff’s reagent (5 min), sulphite water (0.5 g sodium disulfate in 100 ml distilled water; 2 × 3 min) and running water (10 min). After washing in distilled water, hematoxylin (VWR International, Dublin, Ireland; 30 s) was applied as a counterstain. After dehydration in ascending series of ethanols (70%, 80%, 100%, 100%; 5 min each), slides were coverslipped with Dako mounting medium (Agilent Technologies, Co. Cork, Ireland). Glycogen and glycoproteins synthesized in the tubal epithelium were discriminated by digestion of glycogen with 1% Amylase (Sigma‐Aldrich; 37 °C, 10 min) before placement in 0.5% periodic acid.

For Alcian blue staining, sections were put into 3% acetic acid (Sigma-Aldrich, Wicklow, Ireland; 3 min), followed by 1% Alcian blue in 3% acetic acid (Sigma-Aldrich, Wicklow, Ireland; pH 2.5, 30 min) and 3% acetic acid (Sigma-Aldrich, Wicklow, Ireland; 3 min). After washing with distilled water (2 × 3 min), sections were counterstained with nuclear factor red (Sigma-Aldrich, Wicklow, Ireland; 5 min). After dehydration in ascending series of ethanols (70–100%), slides were coverslipped as described before. Documentation of the samples was performed using an Olympus BX51 microscope (Olympus, Hamburg, Germany) and a DP71 camera (Masontec, Dublin, Ireland). The area in the tubal epithelium stained positive for glycoproteins and mucopolysaccharides, respectively, was calculated in each sample in 5 ROIs (0.07 mm^2^) using ImageJ.

### Scanning electron microscopy (SEM)

SEM was performed to analyse the tubal morphology and secretory activity before and after sperm binding and to compare the microarchitecture of the head and tail of the conventional and sexed spermatozoa bound in the sperm reservoir. For this purpose, 1 cm pieces of closed ampulla (n = 15) were incubated with a) HEPES buffer – no spermatozoa, b) conventional spermatozoa or c) sexed spermatozoa of the same bulls (n = 5) as described above. After co-incubation, the tissues were fixed in a 2.5% glutaraldehyde solution in Sorenson’s buffer (1:5 (v/v) solution of 0.07 M KH_2_PO_4_ and 0.07 M Na_2_HPO_4_–2H_2_O; 4 °C, 1 h). Samples were then dehydrated in an ascending series of acetones (10%, 20%, 30% 40%, 50% and 60%, 5 min; 70%, 80% and 90%, 1 h; 100%, 12 h). Specimens were dried at critical point with liquid CO_2_ (Union Point Dryer CPD 030; Bal-Tec, Walluf, Germany), Coating was performed with 12 nm gold–palladium using a Union SCD 040 sputtering device (Bal-Tec, Walluf, Germany). Specimens were analysed with a Zeiss DSM 950 electron microscope at magnifications ranging from × 50 to × 8000.

### Transmission electron microscopy (TEM)

TEM was performed to compare the ultrastructure of the head, neck and midpiece of conventional and sexed spermatozoa in the tubal sperm reservoir and to compare the ultrastructure of the epithelial cells after sperm binding. 1 cm pieces of closed ampulla (n = 6) were incubated with conventional or sexed spermatozoa as described above. In control samples, ampullae were incubated with HEPES buffer without spermatozoa. In total, 750 spermatozoa from 3 bulls in 9 ampullae and 750 sperm heads, 124 sperm necks and 173 sperm midpieces with 2717 mitochondria were investigated. The samples were fixed in a 2.5% glutaraldehyde solution in Sorenson’s buffer (1:5 (v/v) solution of 0.07 M KH_2_PO_4_ and 0.07 M Na_2_HPO_4_–2H_2_O; 4 °C, 1 h). After fixation, the samples were cut into 0.4 mm pieces with the end parts of the sample being discarded. After washing in cacodylic acid buffer (0.1 M C_2_H_7_AsO_2,_ 3 × 5 min), specimens were contrasted with osmium tetroxide potassium ferrocyanide (OsO_4_-K_4_Fe(CN)_6_.[Fe(CN_6_)]^4−^, 4 h). After washing in cacodylic acid buffer (OsO_4_-K_4_Fe(CN)_6_.[Fe(CN_6_)]^4−^, RT, 4 h), samples were dehydrated in an ascending series of ethanols (70%, 80%, 90%, 100%, 100%; 2 × 15 min each). They were then placed in a 2:1 ratio solution of propylene oxide (C_3_H_6_O): epoxy resin (1 h), followed by a 1:1 ratio solution of propylene oxide (C_3_H_6_O): epoxy resin (12 h), and a 2:1 ratio solution of propylene oxide (C_3_H_6_O): epoxy resin (1 h). They were then placed in pure epoxy resin. Semi-thin (0.5 µm) sections were cut and stained with methylene blue for initial evaluation. Sections with spermatozoa bound to oviductal epithelial cells were cut into ultrathin (90 nm) sections and placed on copper grids. The ultrathin sections were contrasted with a saturated solution of uranyl acetate (UO_2_(CH_3_COO)_2_.2H_2_O; 10 min). Sections were then washed in dH_2_O and dried. Analyses were performed with a Zeiss EM 902 transmission electron microscope at magnifications of × 5000 to × 30,000. For documentation, the Slow Scan CCD Camera 7899 (TRS, Moorenweis, Germany) and the software Image SP 1.2.8.111 (Sys Prog, min k, Belarus) were applied.

### Statistical analyses

Data were analysed using SPSS 24.0 (IBM, New York, USA). Normality was analysed using the Kolmogorov–Smirnov and Shapiro–Wilk tests. Data for sperm behaviour in the reservoir as well as numerical data from TEM were normally distributed. Data for CBF, PTS and histochemical staining were not normally distributed. Analyses were all performed as two-tailed tests. The area under the curve (AUC) and two-tailed t-test were used to assess differences in agile, lagging, immotile and hyperactivated sperm between conventional and sexed sperm reservoirs. For CBF, the Generalised Estimating Equations procedure was used to assess the effect of sperm binding and sorting. For PTS, a two-tailed Wilcoxon test for paired samples was used to compare the mean ranks between oviducts without spermatozoa, with conventional spermatozoa or with sexed spermatozoa. The areas stained positive for glycoproteins and acidic mucopolysaccharides between oviducts incubated with conventional or sexed spermatozoa were compared using Friedman’s two-way analysis of variance by ranks. In the TEM experiments, differences in the frequency of focal indentations and irregular mitochondria in conventional and sexed spermatozoa were analysed using the Chi Square Test. The odds ratio, which quantifies the strength of the association between the application of the sex-sorting process and the occurrence of cellular alterations in the spermatozoa, was calculated as well. An odds ratio greater than 1 indicates a positive association, whereas an odds ration less than 1 indicates a negative association.

## Supplementary information

Supplementary Legends.

Supplementary Video 1.

Supplementary Video 2.

Supplementary Video 3.

Supplementary Video 4.

Supplementary Video 5.

Supplementary Video 6.

Supplementary Video 7.

Supplementary Video 8.

## Data Availability

The data that support the findings of this study are available from the corresponding author, Prof. Dr. Sabine Kölle, upon reasonable request.

## References

[1] Talevi R, Gualtieri R (2010). Molecules involved in sperm-oviduct adhesion and release. Theriogenology.

[2] Kolle S (2009). Ciliary transport, gamete interaction, and effects of the early embryo in the oviduct: ex vivo analyses using a new digital videomicroscopic system in the cow. Biol. Reprod..

[3] Suarez SS, Pacey AA (2006). Sperm transport in the female reproductive tract. Hum. Reprod. Update.

[4] Ellington JE (1999). In vitro interactions of cryopreserved stallion spermatozoa and oviduct (uterine tube) epithelial cells or their secretory products. Anim. Reprod. Sci..

[5] Rodriguez-Martinez H (2005). Boar spermatozoa in the oviduct. Theriogenology.

[6] Rodriguez-Martinez H (2007). Role of the oviduct in sperm capacitation. Theriogenology.

[7] Hunter RH (2008). Sperm release from oviduct epithelial binding is controlled hormonally by peri-ovulatory graafian follicles. Mol. Reprod. Dev..

[8] Suarez SS (2008). Regulation of sperm storage and movement in the mammalian oviduct. Int. J. Dev. Biol..

[9] Kolle S, Reese S, Kummer W (2010). New aspects of gamete transport, fertilization, and embryonic development in the oviduct gained by means of live cell imaging. Theriogenology.

[10] Suarez S, Redfern K, Raynor P, Martin F, Phillips DM (1991). Attachment of boar sperm to mucosal explants of oviduct in vitro: possible role in formation of a sperm reservoir. Biol. Reprod..

[11] Suarez SS (2002). Formation of a reservoir of sperm in the oviduct. Reprod. Domest. Anim..

[12] Rath D, Schuberth HJ, Coy P, Taylor U (2008). Sperm interactions from insemination to fertilization. Reprod. Domest. Anim..

[13] Lefebvre R, Lo MC, Suarez SS (1997). Bovine sperm binding to oviductal epithelium involves fucose recognition. Biol. Reprod..

[14] Dobrinski I, Ignotz GG, Thomas PG, Ball BA (1996). Role of carbohydrates in the attachment of equine spermatozoa to uterine tubal (oviductal) epithelial cells in vitro. Am. J. Vet. Res..

[15] Machado SA (2014). LewisX-containing glycans on the porcine oviductal epithelium contribute to formation of the sperm reservoir. Biol. Reprod..

[16] Green CE, Bredl J, Holt WV, Watson PF, Fazeli A (2001). Carbohydrate mediation of boar sperm binding to oviductal epithelial cells in vitro. Reproduction.

[17] DeMott RP, Lefebvre R, Suarez SS (1995). Carbohydrates mediate the adherence of hamster sperm to oviductal epithelium. Biol. Reprod..

[18] Pini T (2018). Binder of sperm proteins 1 and 5 have contrasting effects on the capacitation of ram spermatozoa. Biol. Reprod..

[19] Hung PH, Suarez SS (2012). Alterations to the bull sperm surface proteins that bind sperm to oviductal epithelium. Biol. Reprod..

[20] Georgiou AS (2007). Modulation of the oviductal environment by gametes. J. Proteome Res..

[21] Bergqvist AS (2005). Hyaluronan and its binding proteins in the epithelium and intraluminal fluid of the bovine oviduct. Zygote.

[22] Sostaric E (2008). Sperm binding properties and secretory activity of the bovine oviduct immediately before and after ovulation. Mol. Reprod. Dev..

[23] Alminana C (2014). The battle of the sexes starts in the oviduct: modulation of oviductal transcriptome by X and Y-bearing spermatozoa. BMC Genom..

[24] Gualtieri R (2013). Bovine oviductal monolayers cultured under three-dimension conditions secrete factors able to release spermatozoa adhering to the tubal reservoir in vitro. Theriogenology.

[25] Gualtieri R, Mollo V, Duma G, Talevi R (2009). Redox control of surface protein sulphhydryls in bovine spermatozoa reversibly modulates sperm adhesion to the oviductal epithelium and capacitation. Reproduction.

[26] Talevi R, Gualtieri R (2001). Sulfated glycoconjugates are powerful modulators of bovine sperm adhesion and release from the oviductal epithelium in vitro. Biol. Reprod..

[27] Talevi R, Zagami M, Castaldo M, Gualtieri R (2007). Redox regulation of sperm surface thiols modulates adhesion to the fallopian tube epithelium. Biol. Reprod..

[28] Lamy J (2018). Identification by proteomics of oviductal sperm-interacting proteins. Reproduction.

[29] Machado SA, Sharif M, Wang H, Bovin N, Miller DJ (2019). Release of porcine sperm from oviduct cells is stimulated by progesterone and requires CatSper. Sci. Rep..

[30] Suarez SS (2016). Mammalian sperm interactions with the female reproductive tract. Cell Tissue Res..

[31] de Oliveira Carvalho J (2018). Flow cytometry sex sorting affects bull sperm longevity and compromises their capacity to bind to oviductal cells. Livest. Sci..

[32] Carvalho J, Sartori R, Dode M (2014). Different ways to evaluate bovine sexed sperm in vitro. Anim. Reprod..

[33] Butler ST, Hutchinson IA, Cromie AR, Shalloo L (2014). Applications and cost benefits of sexed semen in pasture-based dairy production systems. Animal.

[34] Raddatz MS (2008). Enrichment of cell-targeting and population-specific aptamers by fluorescence-activated cell sorting. Angew. Chem. Int. Ed. Engl..

[35] DeJarnette JM, Nebel RL, Marshall CE (2009). Evaluating the success of sex-sorted semen in US dairy herds from on farm records. Theriogenology.

[36] Bodmer M (2005). Fertility in heifers and cows after low dose insemination with sex-sorted and non-sorted sperm under field conditions. Theriogenology.

[37] Seidel GE, Schenk JL (2008). Pregnancy rates in cattle with cryopreserved sexed sperm: effects of sperm numbers per inseminate and site of sperm deposition. Anim. Reprod. Sci..

[38] O'Brien JK (2005). Flow cytometric sorting of fresh and frozen-thawed spermatozoa in the western lowland gorilla (Gorilla gorilla gorilla). Am. J. Primatol..

[39] O'Brien JK, Robeck TR (2006). Development of sperm sexing and associated assisted reproductive technology for sex preselection of captive bottlenose dolphins (Tursiops truncatus). Reprod. Fertil. Dev..

[40] Johnson LA, Rath D, Vazquez JM, Maxwell WM, Dobrinsky JR (2005). Preselection of sex of offspring in swine for production: current status of the process and its application. Theriogenology.

[41] Buchanan BR (2000). Insemination of mares with low numbers of either unsexed or sexed spermatozoa. Theriogenology.

[42] de Graaf SP, Evans G, Maxwell WM, Cran DG, O’Brien JK (2007). Birth of offspring of pre-determined sex after artificial insemination of frozen-thawed, sex-sorted and re-frozen-thawed ram spermatozoa. Theriogenology.

[43] Barros Mothe G (2018). Sperm sexing with density gradient centrifugation in dogs. Anim. Reprod. Sci..

[44] Karabinus DS (2014). The effectiveness of flow cytometric sorting of human sperm (MicroSort(R)) for influencing a child's sex. Reprod. Biol. Endocrinol..

[45] Johnson LA, Flook JP, Look MV, Pinkel D (1987). Flow sorting of X and Y chromosome-bearing spermatozoa into two populations. Gamete Res..

[46] Johnson LA, Welch GR, Rens W (1999). The Beltsville sperm sexing technology: high-speed sperm sorting gives improved sperm output for in vitro fertilization and AI. J. Anim. Sci..

[47] Cottle DJ, Wallace M, Lonergan P, Fahey AG (2018). Bioeconomics of sexed semen utilization in a high-producing Holstein-Friesian dairy herd. J. Dairy Sci..

[48] Frijters AC (2009). What affects fertility of sexed bull semen more, low sperm dosage or the sorting process?. Theriogenology.

[49] Andersson M, Taponen J, Kommeri M, Dahlbom M (2006). Pregnancy rates in lactating Holstein-Friesian cows after artificial insemination with sexed sperm. Reprod. Domest. Anim..

[50] Maicas C (2019). Fertility of fresh and frozen sex-sorted semen in dairy cows and heifers in seasonal-calving pasture-based herds. J. Dairy Sci..

[51] Mallory DA, Lock SL, Woods DC, Poock SE, Patterson DJ (2013). Hot topic: comparison of sex-sorted and conventional semen within a fixed-time artificial insemination protocol designed for dairy heifers. J. Dairy Sci..

[52] Steele H, Makri D, Maalouf WE, Reese S, Kolle S (2020). Bovine sperm sexing alters sperm morphokinetics and subsequent early embryonic development. Sci. Rep..

[53] Balao de Silva CM, Ortega-Ferrusola C, Morrell JM, Rodriguez Martinez H, Pena FJ (2016). Flow cytometric chromosomal sex sorting of stallion spermatozoa induces oxidative stress on mitochondria and genomic DNA. Reprod. Domest. Anim..

[54] Balaoda Silva CM (2013). Sex sorting increases the permeability of the membrane of stallion spermatozoa. Anim. Reprod. Sci..

[55] Baker MA, Aitken RJ (2005). Reactive oxygen species in spermatozoa: methods for monitoring and significance for the origins of genetic disease and infertility. Reprod. Biol. Endocrinol..

[56] Boe-Hansen GB, Morris ID, Ersboll AK, Greve T, Christensen P (2005). DNA integrity in sexed bull sperm assessed by neutral Comet assay and sperm chromatin structure assay. Theriogenology.

[57] de Graaf SP, Evans G, Maxwell WM, O’Brien JK (2006). In vitro characteristics of fresh and frozen-thawed ram spermatozoa after sex sorting and re-freezing. Reprod. Fertil. Dev..

[58] Hollinshead FK, Gillan L, O'Brien JK, Evans G, Maxwell WM (2003). In vitro and in vivo assessment of functional capacity of flow cytometrically sorted ram spermatozoa after freezing and thawing. Reprod. Fertil. Dev..

[59] Carvalho JO, Sartori R, Machado GM, Mourao GB, Dode MA (2010). Quality assessment of bovine cryopreserved sperm after sexing by flow cytometry and their use in in vitro embryo production. Theriogenology.

[60] Holden SA (2017). In vitro characterisation of fresh and frozen sex-sorted bull spermatozoa. Reprod. Fertil. Dev..

[61] Owhor LE, Reese S, Kolle S (2019). Salpingitis impairs bovine tubal function and sperm-oviduct interaction. Sci. Rep..

[62] Noreikat K, Wolff M, Kummer W, Kolle S (2012). Ciliary activity in the oviduct of cycling, pregnant, and muscarinic receptor knockout mice. Biol. Reprod..

[63] Kolle S (2012). Live cell imaging of the oviduct. Methods Enzymol..

[64] Ardon F (2016). Dynamics of bovine sperm interaction with epithelium differ between oviductal isthmus and ampulla. Biol. Reprod..

[65] Bongso A (1989). Establishment of human ampullary cell cultures. Hum. Reprod..

[66] Gwathmey TM, Ignotz GG, Mueller JL, Manjunath P, Suarez SS (2006). Bovine seminal plasma proteins PDC-109, BSP-A3, and BSP-30-kDa share functional roles in storing sperm in the oviduct. Biol. Reprod..

[67] Defaus S, Aviles M, Andreu D, Gutierrez-Gallego R (2016). Identification of bovine sperm surface proteins involved in carbohydrate-mediated fertilization interactions. Mol. Cell Proteom..

[68] Machado-Oliveira G (2008). Mobilisation of Ca2+ stores and flagellar regulation in human sperm by S-nitrosylation: a role for NO synthesised in the female reproductive tract. Development.

[69] Steinhauser CB, Graham JK, Lenz RW, Seidel GE (2016). Removing seminal plasma improves bovine sperm sex-sorting. Andrology.

[70] Druart X (2012). Sperm interaction with the female reproductive tract. Reprod. Domest. Anim..

[71] Gualtieri R, Talevi R (2000). In vitro-cultured bovine oviductal cells bind acrosome-intact sperm and retain this ability upon sperm release. Biol. Reprod..

[72] Ho HC, Suarez SS (2001). Hyperactivation of mammalian spermatozoa: function and regulation. Reproduction.

[73] Taylor JF, Schnabel RD, Sutovsky P (2018). Identification of genomic variants causing sperm abnormalities and reduced male fertility. Anim. Reprod. Sci..

[74] Liao SB, Ho JC, Tang F (2011). Adrenomedullin increases ciliary beat frequency and decreases muscular contraction in the rat oviduct. Reproduction.

[75] Morales P, Palma V, Salgado AM, Villalon M (1996). Sperm interaction with human oviductal cells in vitro. Hum. Reprod..

[76] O'Doherty AM, Di Fenza M, Kolle S (2016). Lipopolysaccharide (LPS) disrupts particle transport, cilia function and sperm motility in an ex vivo oviduct model. Sci. Rep..

[77] Bylander A (2010). Rapid effects of progesterone on ciliary beat frequency in the mouse fallopian tube. Reprod. Biol. Endocrinol..

[78] Halbert SA, Tam PY, Blandau RJ (1976). Egg transport in the rabbit oviduct: the roles of cilia and muscle. Science.

[79] Halbert SA, Becker DR, Szal SE (1989). Ovum transport in the rat oviductal ampulla in the absence of muscle contractility. Biol. Reprod..

[80] Afzelius BA, Camner P, Eliasson R, Mossberg B (1978). Kartagener's syndrome does exist. Lancet.

[81] Pedersen M (1983). Specific types of abnormal ciliary motility in Kartagener's syndrome and analogous respiratory disorders. A quantified microphoto-oscillographic investigation of 27 patients. Eur. J. Respir. Dis. Suppl..

[82] Nosrati R, Graham PJ, Liu Q, Sinton D (2016). Predominance of sperm motion in corners. Sci. Rep..

[83] Romero-Aguirregomezcorta J, Sugrue E, Martinez-Fresneda L, Newport D, Fair S (2018). Hyperactivated stallion spermatozoa fail to exhibit a rheotaxis-like behaviour, unlike other species. Sci. Rep..

[84] Guidobaldi HA (2015). Disrupting the wall accumulation of human sperm cells by artificial corrugation. Biomicrofluidics.

[85] Xu C, Bailly-Maitre B, Reed JC (2005). Endoplasmic reticulum stress: cell life and death decisions. J. Clin. Invest..

[86] Alminana C, Bauersachs S (2019). Extracellular vesicles in the oviduct: progress, challenges and implications for the reproductive success. Bioengineering (Basel).

[87] Pillai VV, Weber DM, Phinney BS, Selvaraj V (2017). Profiling of proteins secreted in the bovine oviduct reveals diverse functions of this luminal microenvironment. PLoS ONE.

[88] Cerdeira J (2020). Cryopreservation effects on canine sperm morphometric variables and ultrastructure: comparison between vitrification and conventional freezing. Cryobiology.

[89] Agarwal A, Varghese AC, Sharma RK (2009). Markers of oxidative stress and sperm chromatin integrity. Methods Mol. Biol..

[90] Nagata O (2018). Mouse model of chorea-acanthocytosis exhibits male infertility caused by impaired sperm motility as a result of ultrastructural morphological abnormalities in the mitochondrial sheath in the sperm midpiece. Biochem. Biophys. Res. Commun..

[91] Amaral A, Lourenco B, Marques M, Ramalho-Santos J (2013). Mitochondria functionality and sperm quality. Reproduction.

[92] Li CY (2018). Resveratrol significantly improves the fertilisation capacity of bovine sex-sorted semen by inhibiting apoptosis and lipid peroxidation. Sci. Rep..

[93] Shang Y (2017). Essential role for SUN5 in anchoring sperm head to the tail. Elife.

[94] Vishwanath R, Moreno JF (2018). Review: semen sexing—current state of the art with emphasis on bovine species. Animal.

[95] Johnson LA, Flook JP, Look MV (1987). Flow cytometry of X and Y chromosome-bearing sperm for DNA using an improved preparation method and staining with Hoechst 33342. Gamete Res..

