# Supplementary material for: Bovine sperm-oviduct interactions are characterized by specific sperm behaviour, ultrastructure and tubal reactions which are impacted by sex sorting
Source: Sci Rep. 2020 Oct 5;10:16522. doi: 10.1038/s41598-020-73592-1 (PMC7536416; doi:10.1038/s41598-020-73592-1)
Supplement: Supplementary file 1 — Supplementary Legends. [file 41598_2020_73592_MOESM1_ESM.docx]

**Bovine Sperm-Oviduct Interactions are Characterized by Specific Sperm Behaviour, Ultrastructure and Tubal Reactions Which are Impacted by Sex Sorting**

Miguel Camara Pirez^1^, Heather Steele^1^, Sven Reese^2^ & Sabine Kölle^1*^

^1^School of Medicine, Health Sciences Centre, University College Dublin (UCD), Dublin, Ireland. ^2^ School of Veterinary Medicine, Institute of Veterinary Anatomy, Histology and Embryology, LMU, Munich, Germany.

**Supplementary Information Guide**

**Movie 1** Bovine sperm binding and formation of the conventional sperm reservoir in the ampulla

**Movie 2** Binding of sex-sorted sperm in the ampulla

**Movie 3** Vital sperm binding in a tangential angle (2) as well as immotile (1) and hyperactive sperm (3)

**Movie 4** Lagging sex-sorted sperm in the ampulla

**Movie 5** Rotational movements of sperm after sex sorting

**Movie 6** Immotile sperm in the ampulla after sex-sorting

**Movie 7** Hyperactive sperm in the oviduct

**Movie 8** Measurement of CBF (Part 1) and PTS (Part 2) in the bovine ampulla
